# Emergence of spatially heterogeneous burst suppression in a neural field model of electrocortical activity

**DOI:** 10.3389/fnsys.2015.00018

**Published:** 2015-02-26

**Authors:** Ingo Bojak, Zhivko V. Stoyanov, David T. J. Liley

**Affiliations:** ^1^Systems Neuroscience Research Group, School of Systems Engineering, University of ReadingReading, UK; ^2^Brain and Psychological Sciences Research Centre, School of Health Sciences, Swinburne University of TechnologyHawthorn, VIC, Australia

**Keywords:** burst suppression, anesthesia, EEG, neural field model, neuronal hyperexcitability

## Abstract

Burst suppression in the electroencephalogram (EEG) is a well-described phenomenon that occurs during deep anesthesia, as well as in a variety of congenital and acquired brain insults. Classically it is thought of as spatially synchronous, quasi-periodic bursts of high amplitude EEG separated by low amplitude activity. However, its characterization as a “global brain state” has been challenged by recent results obtained with intracranial electrocortigraphy. Not only does it appear that burst suppression activity is highly asynchronous across cortex, but also that it may occur in isolated regions of circumscribed spatial extent. Here we outline a realistic neural field model for burst suppression by adding a slow process of synaptic resource depletion and recovery, which is able to reproduce qualitatively the empirically observed features during general anesthesia at the whole cortex level. Simulations reveal heterogeneous bursting over the model cortex and complex spatiotemporal dynamics during simulated anesthetic action, and provide forward predictions of neuroimaging signals for subsequent empirical comparisons and more detailed characterization. Because burst suppression corresponds to a dynamical end-point of brain activity, theoretically accounting for its spatiotemporal emergence will vitally contribute to efforts aimed at clarifying whether a common physiological trajectory is induced by the actions of general anesthetic agents. We have taken a first step in this direction by showing that a neural field model can qualitatively match recent experimental data that indicate spatial differentiation of burst suppression activity across cortex.

## 1. Introduction

Over the many years since its discovery in humans (Berger, 1929, 1930; Adrian and Matthews, 1934), the electroencephalogram (EEG) has been shown to be a sensitive, and often specific, indicator of brain state and function (Schomer and Lopes da Silva, 2010). In the case of the deeply inactivated brain, whether through trauma or medical intervention, a burst suppression pattern is typically observed (Niedermeyer, 2009; Ching et al., 2012). Consisting of quasi-periodic alternations of high amplitude periods of spiking activity with low amplitude periods that are near isoelectric, the burst suppression pattern is associated with a range of central insults or interventions that include cortical deafferentation (Henry and Scoville, 1952; Kellaway et al., 1966; Lukatch and MacIver, 1996), cerebral ischaemia (Bauer et al., 2013), deep coma (Young, 2000), various infantile encephalopathies (Grigg-Damberger et al., 1989), the final stages of deteriorated status epilepticus (Treiman et al., 1990), hypothermia (Stecker et al., 2001), and high levels of many anesthetic and sedative drugs (Schwartz et al., 1989; Akrawi et al., 1996).

The burst suppression pattern can show a significant degree of variation depending on its aetiology. For example, in the case of infantile hypoxic-ischemic encephalopathy the burst suppression pattern can be quite complex; and due to significant variability in the amplitude of individual bursts a clear transition to suppression may not readily be apparent (Lamblin et al., 2013). In contrast, in deep anesthesia bursts are typically separated by clear isoelectric periods, the duration (relative and absolute) of which increases systematically with increasing anesthetic level. This systematic dependence on anesthetic level can be utilized in the treatment of status epilepticus (Kalviainen et al., 2005) and the management of brain trauma in the intensive care setting (Doyle and Matta, 1999) by defining an endpoint in which more than 50% of an EEG recording consists of suppressions.

In what follows we will provide an overview of the phenomenon of burst suppression and summarize the current understanding regarding its physiological genesis. This will then be followed by a detailed outline of a neural field model developed to describe the emergence of burst suppression during anesthesia, which notably, and for the first time, incorporates the empirically realistic modeling of a general anesthetic agent (isoflurane) and the spatio-temporal propagation of cortical activity.

### 1.1. Physiological basis of burst suppression

Despite its clear aetiological associations and clinical utility, little is known about the physiological mechanisms responsible for the genesis of burst suppression (Liley and Walsh, 2013). On the basis of brain slice and *in vivo* animal studies, a number of hypotheses have been advanced with sometimes contradictory conclusions. For example both increases (Steriade et al., 1994) and decreases (Ferron et al., 2009) in GABAergic inhibitory activity have been speculated to have causal roles in the onset of burst suppression. Supporting reductions in inhibition are *in vivo* whole-brain animal studies suggesting that enhanced network excitability (Detsch et al., 2002; Hudetz and Imas, 2007; Kroeger and Amzica, 2007; Land et al., 2012), possibly mediated through alterations in extracellular calcium (Kroeger and Amzica, 2007), is responsible for driving transitions between low amplitude quiescence and high amplitude bursting. The study of Land et al. (2012) is particularly relevant in this regard. Not only do they report that auditory and visual stimuli readily evoke burst activity in visual cortex (V1) and subiculum during deep anesthesia in rats, but (i) such excitability does not occur in the absence of burst suppression, (ii) V1 and subiculum bursting, in response to the cortically remote auditory stimulus, emerges abruptly with increasing anesthetic (isoflurane) concentration, and (iii) hysteresis occurs in both stimulus-induced and spontaneous bursting during isoflurane wash-in and wash-out. Thus, the phenomenon of burst suppression might be explicable in terms of the emergence of propagating excitability through a dynamical bifurcation parametrically regulated by isoflurane concentration.

Clinically it is well-established that bursting responses during burst suppression in deep anesthesia can be readily evoked by noxious and sensory stimulation, thus further implicating a role for alterations in cortical excitability in the genesis of burst suppression. For example, in probably the first systematic study on evoked bursts, Yli-Hankala et al. (1993) observed that a vibratory stimulus applied to the palm of the hand was readily able to evoke electroencephalographic bursts in patients during moderately deep isoflurane anesthesia. Subsequently it has been found that a range of visual, auditory, tactile and noxious stimuli are able to evoke electroencephalographic bursts during deep anesthesia in which burst suppression has been variously induced with isoflurane (Hartikainen et al., 1995), sevoflurane (Jantti et al., 1998) or propofol (Huotari et al., 2004).

Complementing this empirical and clinical research are recent modeling studies (Ching et al., 2012; Liley and Walsh, 2013), which suggest that the onset or unmasking of slow and activity-dependent modulations of network excitability might account for burst suppression patterns. Because it is observed that the spectral characteristics of the EEG just prior to the onset of the anesthesia-induced burst suppression are conserved in the bursts^1^, such theoretical approaches typically modulate the oscillatory system that accounts for the dynamical emergence of the resting and anesthetic EEG. In order to simulate burst suppression during deep propofol anesthesia, Ching et al. (2012) utilize a thalamo-cortical model based on individual neurons previously developed to account for the propofol-induced emergence of frontal alpha-spindle activity (Ching et al., 2010). This model is then augmented with a slow adenosine triphosphate (ATP) gated potassium membrane current, which is hence regulated by the activity-dependent metabolic production rate of ATP. By assuming that propofol down-regulates neuronal firing through enhanced synaptic inhibition, thus leading to an autoregulatory decrease in cerebral metabolism and hence ATP production, the modulatory effect of this potassium current is magnified such that bursting emerges. In contradistinction to this model, in which bursting arises due to essentially intrinsic changes of neuronal excitability, Liley and Walsh (2013) developed a model in which bursting arises as a consequence of the slow activity-dependent modulation of synaptic efficacy. In this model the effects of synaptic resource depletion (receptor desensitization and synaptic vesicle depletion) and recovery during periods of sustained neuronal population activity act to slowly modulate neuronal population excitability. This mechanism comes to the fore in anesthesia because the general reduction of cortical activity allows the synaptic neurotransmitter reservoirs to fill up, potentiating excitation until it is sufficiently strong to induce feedback bursts of excitation, followed by suppression as the thereby depleted reservoirs refill. In support of this mechanism are the activity-dependent alterations in synaptic efficacy that have been been observed *in vivo* in recordings in cats during burst suppression induced with isoflurane (Kroeger and Amzica, 2007).

### 1.2. Spatio-temporal features of burst suppression

Because burst suppression is classically characterized as being a spatially homogeneous phenomenon (Brenner, 1985; An et al., 1996; Lewis et al., 2013), on the basis of near simultaneous burst onset and offset across scalp electrode derivations, little attention has been paid to its spatio-temporal features until recently. Motivated by the inability of scalp electroencephalography to reveal the fine structure of cortical dynamics, due to the spatial blurring induced by volume conduction, Lewis et al. (2013) chose to investigate the spatiotemporal features of burst suppression using intracranial electrocortigraphy (ECoG) in medically intractable epilepsy patients. Five patients, implanted with a range of subdural strip, grid and depth electrodes as part of a standard clinical monitoring procedure, had recordings collected throughout the induction of anesthesia with propofol, during explantation surgery. Burst onset and offset was observed to be visibly asynchronous across recording electrodes, with the absolute difference in burst onset time in general an increasing function of inter-electrode distance. Interestingly, not all recording electrodes would participate in such asynchronous bursting. It was found that burst onsets were visibly clustered across channels such that bursting could either be confined to a small subset of nearby electrodes (“local” bursting) or spread to involve the whole electrode grid (“global” bursting), with more distantly separated electrode pairs less likely to share a burst (based on burst onset within some time window).

It has been speculated that the appearance of spatially inhomogeneous bursting might be a reflection of the differential sensitivity of specific thalamo-cortical networks to anesthetic action. However, another possibility is that the spatially heterogeneous nature of this bursting arises as a feature of the axonal propagation of activity through cortex. In support of such a speculation is the developmental emergence of isoflurane-induced burst suppression in rats. It is conjectured that it is the development of short- and long-range horizontal connections between pyramidal neurons in infra-granular cortical layers, which is the critical factor in determining the appearance of isoflurane-induced burst suppression in the second postnatal week (Sitdikova et al., 2014). Further implicating the role that altered propagation may have in determining the physiological features of anesthetic action are reports that document the effects anesthetics have on nerve conduction—both centrally and peripherally. While peripherally it is generally assumed that anesthetics principally depress spinal motoneuron excitability, as assessed by reductions in F-wave amplitudes (Friedman et al., 1996; Rampil and King, 1996), there are a number of reports documenting the significant effects of anesthetic agents in either increasing (cyclopropane, nitrous oxide, diethyl ether) (Rosner et al., 1971) or reducing (pentobarbital, desflurane, enflurane, halothane) (Rampil and King, 1996; Oh et al., 2010; Nowicki et al., 2013) nerve conduction velocity at clinical levels, as assessed by increases in F-wave latency. Centrally, there is some evidence that volatile anesthetics may preferentially depress nerve conduction in unmyelinated axons (Berg-Johnsen and Langmoen, 1986; Mikulec et al., 1998). For instance, isoflurane was found to induce a conduction block in 20–30% of the unmyelinated fibers in the CA1 region of the rat hippocampus at clinical concentrations, as well as having a 1% effect on the actual conduction velocity (Berg-Johnsen and Langmoen, 1986). On the basis of empirical evidence indicating that the cortico-cortical fiber system is comprised of a mixture of myelinated and unmyelinated fibers, cf. Bojak and Liley (2010) and references therein, we hence expect mean cortical axonal conduction velocity to increase slightly, due to the reduction in the proportion of low conduction velocity unmyelinated fibers, but nevertheless anticipate cortico-cortical synaptic connectivity to be attenuated.

### 1.3. Necessity of large-scale cortical models

Regardless of the specific changes in cortical axonal conduction induced by anesthetics, it is clear that any theoretical attempt to account for burst suppression and its spatial inhomogeneity must explicitly incorporate the spatial extent of cortex. While the construction of a biophysically-based neuronal network model might seem an obvious starting point, numerical tractability and parametric uncertainties militates against the utility of this approach both from a descriptive and an explanatory perspective. For example, to meaningfully accommodate the extent of the spatially heterogeneous burst suppression seen in Lewis et al. (2013), we would need to model ~ 10^9^ neurons and ~ 10^12^ synapses. While computations at this scale may be at the edge of feasibility for the largest supercomputers, we cannot reasonably expect such massive computations to be used for all the myriad specific research agendas in computational neuroscience any time soon. Even if such resources were readily available we would still be unable to specify the microcircuitry realistically at this level of detail for such a sizeable part of cortex. Such a problem will persist even if our computational capabilities continue to grow exponentially.

Fortunately, by considering the behavior of *populations* of neurons at mesoscopic scales, a variety of numerically tractable modeling approaches can be motivated physiologically and anatomically, cf. the reviews of Deco et al. (2008), Coombes (2010), Liley et al. (2012), and Liley (2013). These *neural population models*, referred to as neural mass models if localized and neural field or mean field models if spatially continuous and extensive, usually aim to describe the dynamical evolution of mean quantities (such as soma membrane potential or firing rate) defined over some suitable spatial domain or scale. Because these models average the activity of many thousands of neurons, they are well-suited as frameworks for understanding the meso- and macroscopic neural activity recorded, or inferred by, ECoG, EEG, magnetoencephalography (MEG) and the blood-oxygen level dependent (BOLD) contrast of functional magnetic resonance imaging (fMRI) (Bojak and Breakspear, 2013). Since the pioneering work of Walter Freeman (Freeman, 1975), this approach has flourished and has resulted in a number of important neural field models aimed at explaining the dynamical genesis of the mammalian EEG (Wilson and Cowan, 1973; Lopes da Silva et al., 1974; Nunez, 1974; Liley et al., 2002; Robinson et al., 2004). Broadly speaking, all these models are able to generate oscillatory activity through reverberant feedforward and feedback synaptic activity between excitatory and inhibitory neuronal populations. We choose to utilize the neural field model of Liley et al. (2002) as a framework for better understanding the spatial heterogeneity of bursting during anesthesia because (i) it has been previously employed to account for a number of anesthetic induced EEG changes (Steyn-Ross et al., 1999; Bojak and Liley, 2005), and (ii) a spatially homogeneous version of the theory has been shown to burst when modified to include a slow modulatory system (Liley and Walsh, 2013).

## 2. Neural field model for spatiotemporal burst suppression

Here we detail how a neural field model (Liley et al., 2002), subsequently extended to account for the dynamical genesis of the resting EEG and its modulation by anesthesia (Bojak et al., 2004; Bojak and Liley, 2005; Liley and Bojak, 2005; Frascoli et al., 2011; Liley et al., 2011; Bojak et al., 2013), can be plausibly modified to produce bursting-like behavior (Liley and Walsh, 2013), and thus serve as a basis for understanding the emergence of spatially heterogeneous burst suppression seen in cortex. The main advance in this work is that we combine the realistic modeling of isoflurane effects and the extension to a two-dimensional spatial sheet of Bojak and Liley (2005) with an updated version of the slow modulatory system proposed in Liley and Walsh (2013) in order to obtain spatiotemporal activity predictions.

It is perhaps useful to discuss two fundamental limitations of our current approach in advance. First, we are limiting ourselves here to a two-dimensional (toroidal) cortical sheet and use “background” (isotropic and homogeneous) connectivity in order to use computationally efficient activity propagation with partial differential equations (PDEs). Currently there exist a range of mesoscopic approaches available that can incorporate more realistic cortical geometry as well as including “specific” (anisotropic and sparse) connectivity, see for example (Bojak et al., 2010, 2011; Deco et al., 2011; Bojak and Breakspear, 2013; Sanz Leon et al., 2013) and references therein. These typically involve constructing meshes of neural masses and tracking their information exchanges individually. However, such approaches are computationally about an order of magnitude more expensive. Furthermore, to effectively display spatiotemporal pattern formation on a realistically folded cortex is a graphical challenge. We will show here that spatial differentiation of activity emerges even if one uses a simple toroidal cortical geometry with isotropic and homogeneous connectivity. The additional complexity introduced by anatomical folding structures and patchy connectivity are expected to break up long-range coherence further, but should not qualitatively change our results more locally (where the “background” connectivity is a good approximation) and between well-connected but separated regions (where we would expect emergent differentiation). In addition, we use here the well-known “damped wave” propagation PDEs that have been the mainstay of the field since their initial introduction by Jirsa and Haken (1996). It is by now known that one can use “dispersive” propagation PDEs that are more faithful to the actual distribution of axonal fiber velocities (Bojak and Liley, 2010). However, we are using here a parameter set of Bojak and Liley (2005) that delivers realistic EEG activity under the assumption of “damped wave” propagation. Furthermore, the better “dispersive” propagation is also computationally considerably more expensive and technically difficult to implement. Finally, one of the key results of Bojak and Liley (2010) was that more realistic “dispersive” propagation lead to easier spatiotemporal pattern formation. Thus, we expect that the results here would carry over qualitatively to more realistic propagation models, likely showing spatial differentiation earlier on in the burst phase. In summary, we will show here with the computationally simplest model that spatial differentiation in the burst phase can emerge in qualitative agreement with the experimental observation, and we expect that even more realistic modeling will only enhance these emergent effects.

### 2.1. The (extended) liley model

**Figure 1:**
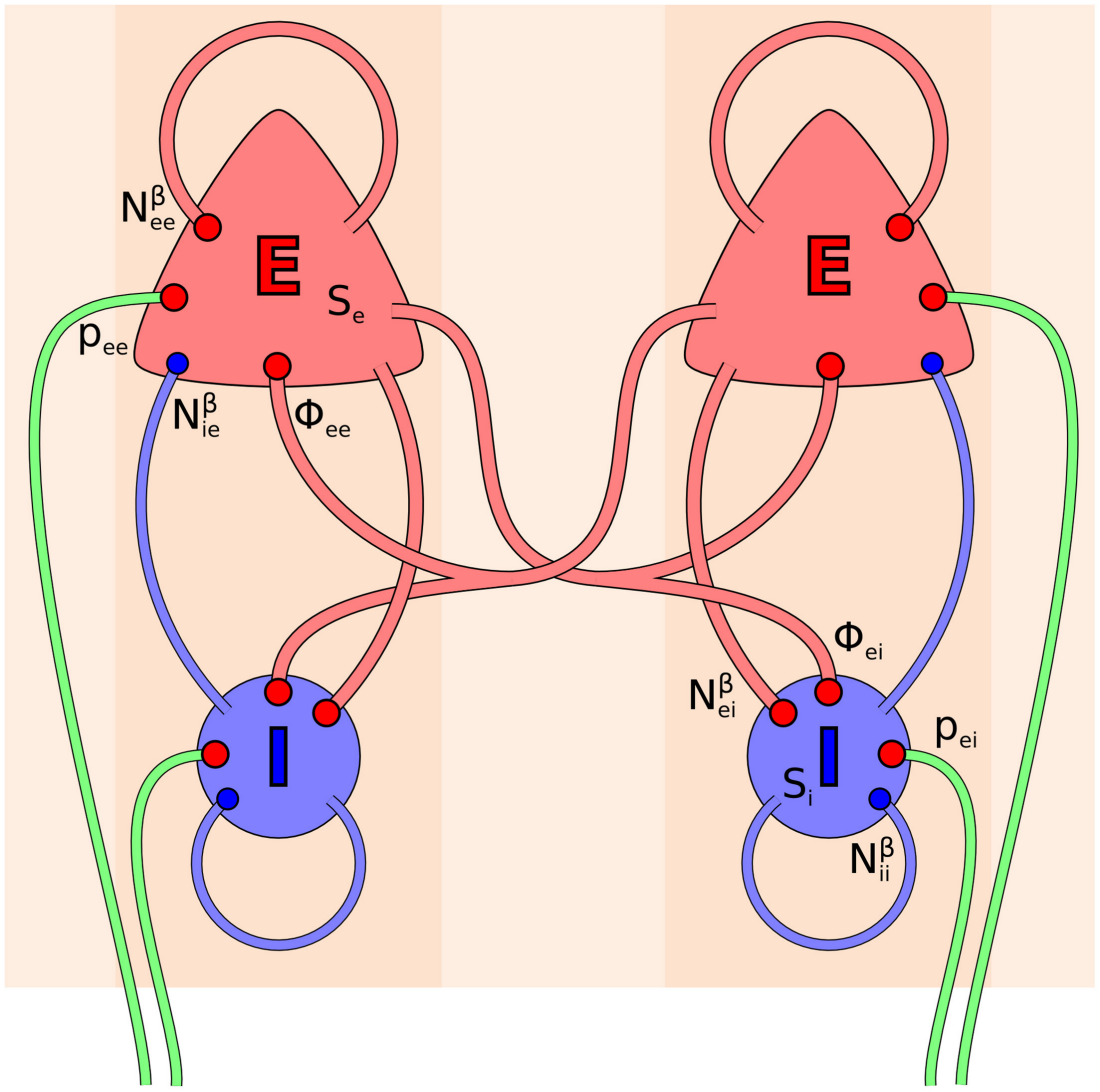
**Topology of the Liley model (Liley et al., 2002; Bojak and Liley, 2005)**. Its two distinct neural populations (E = excitatory, I = inhibitory) are shown for two separate positions on the cortical sheet. Each one can be considered as representing a single macrocolumn. All synaptic connections that occur in the model are shown by red (excitatory) and blue (inhibitory) disks, respectively. Extracortical inputs to the cortical populations are shown by green fibers. Symbols illustrate the various inputs to the excitatory population in the left macrocolumn and to the inhibitory population in the right macrocolumn, respectively, according to Equations (3, 4).

The electrocortical model of Liley et al. (2002) is constructed at the scale of the cortical macrocolumn. Within each macrocolumn, and extending across all cortical layers, distributed populations of excitatory and inhibitory neurons interact with each other by all possible feedforward and feedback intracortical (local) axo-dendritic connections. Macrocolumns then interact with each other by the exclusively excitatory cortico-cortical (long-range) axonal fibers. The topological organization of this model is well-known, and depicted in Figure 1. In this model cortical activity is described by the spatiotemporal evolution of the mean excitatory *h*_*e*_($\vec{x}$, *t*) and inhibitory *h*_*i*_($\vec{x}$, *t*) soma membrane potentials. The connection with electrophysiological measurement is through *h*_*e*_, which is assumed to be linearly related to the EEG, cf. Bojak and Breakspear (2013). Excitatory and inhibitory neuronal populations are modeled as spatially averaged *conductance-based* neurons:

(1) $$\begin{array}{l} \tau_{k}\frac{\partial h_{k}\left(\vec{x},t\right)}{\partial t}=h_{k}^{\text{r}}-h_{k}\left(\vec{x},t\right)+\\ \;\sum_{l\;=\;e,i}\frac{h_{lk}^{\text{eq}}-h_{k}\left(\vec{x},t\right)}{\left|h_{lk}^{\text{eq}}-h_{k}^{\text{r}}\right|}I_{lk}\left(\vec{x},t\right)\;, \end{array}$$

where $\vec{x}$ ∈ ℝ^2^ is position on the cortical sheet, subscripts *l*, *k* ∈ {*e*, *i*} indicate excitatory and inhibitory subpopulations, respectively, and double subscripts represent first the pre-synaptic source and then the post-synaptic target. The parameters *h*^r^_*k*_ are the mean resting membrane potentials to which the *h*_*k*_ decay exponentially with characteristic time scales τ_*k*_ in the absence of inputs *I*_*lk*_. The fraction in front of the *I*_*lk*_ weighs these inputs, so that the depolarizing effect of additional excitation diminishes linearly and then even becomes hyperpolarizing past the reversal potentials *h*^eq^_*ek*_, and similarly for the hyperpolarization due to inhibition depending on *h*^eq^_*ik*_. The weight at the resting potentials is +1 for excitatory and −1 for inhibitory inputs, respectively.

The dynamics of the post-synaptic potentials (PSPs) *I*_*lk*_ are described by critically damped oscillators driven by the mean rate of incoming excitatory or inhibitory axonal pulses *A*_*lk*_, originally defined as follows (Liley et al., 2002):

(2) $${\left(\frac{1}{\gamma_{lk}}\frac{\partial }{\partial t}+1\right)}^{2}I_{lk}\left(\vec{x},t\right)=\frac{e\Gamma_{lk}}{\gamma_{lk}}A_{lk}\left(\vec{x},t\right)\;,$$

(3) $$\begin{array}{l} A_{ek}\left(\vec{x},t\right)=N_{ek}^{\beta }S_{e}\left[h_{e}\left(\vec{x},t\right)\right]\\ \;+N_{ek}^{\alpha }\Phi_{ek}\left(\vec{x},t\right)+p_{ek}\left(\vec{x},t\right)\;, \end{array}$$

(4) $$A_{ik}\left(\vec{x},t\right)=N_{ik}^{\beta }S_{i}\left[h_{i}\left(\vec{x},t\right)\right]\;.$$

For excitatory post-synaptic conductances there are three sources of axonal pulses—local (*S*_*e*_), cortico-cortical (Φ_*ek*_), and subcortical (*p*_*ek*_)—whereas for inhibitory post-synaptic conductances the only source of axonal pulses is local (*S*_*i*_), because thalamic and cortical inhibitory axons are essentially short-range on the basis of existing neuroanatomical evidence. For these equations, at a given location a single pre-synaptic (Dirac delta) spike *A*_*lk*_(*t*) = δ(*t*) would produce a so-called “alpha function” response

(5) $$I_{lk}(t)=\frac{e\Gamma_{lk}}{\gamma_{lk}}\alpha_{lk}(t)\;,$$

(6) $$\alpha_{lk}(t)=\gamma_{lk}^{2}te^{-\gamma_{lk}t}\Theta (t)\;,$$

with the Heaviside step function Θ(*t*). The alpha function α_*lk*_ is normed to one for integration over time, hence the pre-factor in Equation (5) is proportional to the charge transfer of the induced PSP. Furthermore, δ_*lk*_ = 1/γ_*lk*_ is the characteristic time scale of the PSP's exponential decay. Since *I*_*lk*_(*t* = δ_*lk*_) = Γ_*lk*_ is the maximum amplitude of the PSP, δ_*lk*_ is also the rise time to peak amplitude. Note that since we have collapsed all cortical layers into one sheet without radial extension, this rise time is that of the PSP conducted to the soma rather than at the synapse in the dendritic tree. Conduction through a passive dendritic cable effectively leads to a “flattened” PSP at the soma with lower maximum amplitude and prolonged rise and decay times.

The *S*_*e*_ and *S*_*i*_ are respectively the local mean excitatory and inhibitory firing rates, and are assumed to be instantaneous sigmoidal functions of the *h*_*k*_ of the form

(7) $$S_{k}\left[h_{k}\left(\vec{x},t\right)\right]=S_{k}^{\text{max}}/\left\{1+\exp\left[-\sqrt{2}\frac{h_{k}\left(\vec{x},t\right)-\mu_{k}}{\sigma_{k}}\right]\right\}\;,$$

and the *N*^α^_*ek*_ and *N*^β^_*lk*_ factors in the *A*_*lk*_ above multiply these local firing rates by the number of synaptic connections formed with the target populations. The *S*^max^_*k*_ are the maximum mean firing rates, and the μ_*k*_ and σ_*k*_ can be understood as the mean and standard deviation, respectively, of the firing thresholds of the populations, which are taken to be roughly normally distributed. The propagation of axonal pulses by the excitatory cortico-cortical fiber system Φ_*ek*_ is described here by the following well-known “damped wave” equation^2^ (Jirsa and Haken, 1996; Robinson et al., 1997; Liley et al., 2002; Bojak and Liley, 2010):

(8) $$\left[{\left(\frac{1}{v_{ek}}\frac{\partial }{\partial t}+\frac{1}{\lambda_{ek}}\right)}^{2}-\nabla^{2}\right]\Phi_{ek}\left(\vec{x},t\right)=\frac{1}{\lambda_{ek}^{2}}S_{e}\left[h_{e}\left(\vec{x},t\right)\right]\;.$$

But for the λ_*ek*_ terms, this would be an inhomogeneous wave equation with conduction velocity *v*_*ek*_, propagating the local excitatory firing rate *S*_*e*_. However, due to these terms the wave gets suppressed roughly exponentially with distance with a characteristic spatial scale λ_*ek*_.

Finally, there is also extracortical synaptic input in the form of the *p*_*ek*_. These inputs can be considered to be mainly due to thalamic afferents. If the *p*_*ek*_ were constant, then for the model parameters chosen here the system would quickly converge to a static equilibrium point. It is hence the imposition of noise on these inputs which effectively drives the neural activity. This noise is taken to represent the average over the varied extracortical synaptic input to the many thousands of neurons in a neural population, for the case in which there is no strong external (sensory) drive that would lead to clear correlations of the synaptic inputs to the individual neurons. We follow here essentially the approach of Bojak and Liley (2005) for noise generation. Thus, for the sake of computational simplicity noise is imposed only on *p*_*ee*_, whereas *p*_*ei*_ is taken to be constant. At every grid point of the two-dimensional cortical sheet normally distributed noise is generated independently, but with the same mean *p*_*ee*_, and a standard deviation that is 10% of this mean. However, we filter this noise spatiotemporally, both to achieve more biological realism and to make it easier to achieve numerical stability. We follow the Fourier space procedure of Bojak and Liley (2005) for the spatial filtering, but use the Catmull-Rom spline procedure detailed in Bojak et al. (2011) for the temporal filtering, with lowpass −3 dB points at 75 Hz and 2/cm, respectively (Bojak and Liley, 2005). Thus, the noisy input oscillates equally at all frequencies, but only up to about 75 Hz, and is identical for neighboring grid points, but becomes uncorrelated at cortical distances greater than about 0.5 cm. The spatiotemporal *p*_*ee*_ noise breaks the otherwise perfect homogeneity and isotropy of the system, and consequently acts as seed for the heterogeneities observed in the burst suppression phase. However, the characteristics of the spatiotemporal structures that emerge in the burst suppression phase do not otherwise depend on the noise; hence in particular they do not depend on the details of the noise filtering, and can be elicited with white noise driving.

The model we have described so far has to be extended for a realistic description of the effect of general anesthetic action. In particular, the effect of isoflurane on the rise time δ_*lk*_ of the PSPs from zero to maximum amplitude Γ_*lk*_, and on the subsequent decay time ζ_*lk*_ back to Γ_*lk*_/*e* (measured here from the start of the PSP, not from the peak) can be parameterized as follows in the form of a Hill equation (Bojak and Liley, 2005):

(9) $$\delta_{lk}(c)≃\delta_{lk}\;(\text{constant})\;,$$

(10) $$\zeta_{ek}(c)\equiv \zeta_{ek}^{0}\kappa_{ek}(c)≃\zeta_{ek}^{0}\;(\text{constant})\;,$$

(11) $$\zeta_{ik}(c)\equiv \zeta_{ik}^{0}\kappa_{ik}(c)≃\zeta_{ik}^{0}\frac{0.32^{2.7}+4.7c^{2.7}}{0.32^{2.7}+c^{2.7}}\;,$$

where *c* is the aqueous concentration in mM. Thus, the main effect is a prolongation of the decay of the inhibitory PSPs. In addition, the maximum amplitudes of the PSPs also diminish with increased isoflurane concentration, which is also the case for excitatory PSPs:

(12) $$\Gamma_{ek}(c)\equiv \Gamma_{ek}^{0}H_{e}(c)≃\Gamma_{ek}^{0}\frac{0.707^{2.22}}{0.707^{2.22}+c^{2.22}}\;,$$

(13) $$\Gamma_{ik}(c)\equiv \Gamma_{ik}^{0}H_{i}(c)≃\Gamma_{ik}^{0}\frac{0.79^{2.6}+0.56c^{2.6}}{0.79^{2.6}+c^{2.6}}\;.$$

While for consistency with laboratory based estimates we choose to parameterize isoflurane level in terms of its aqueous concentration, it is important to appreciate that because isoflurane is a volatile gas, clinically its level is typically reported in terms of its concentration in the expired air (which is assumed to be in equilibrium with the blood and hence the extracellular fluid of cortical neurons). At normal body temperature 1.3% isoflurane are equivalent to an aqueous concentration of about *c* ≃ 0.27 mM (Franks and Lieb, 1996). Typical isoflurane concentrations encountered in clinical anesthetic practice range from 0 to 2% of the expired air, equivalent to aqueous concentrations 0 − 0.42 mM (Mapleson, 1996). A measure commonly employed in anesthetic practice is the minimum alveolar concentration or MAC of an anesthetic agent. It is essentially defined as the concentration of gas in the lungs required to prevent movement in 50% of subjects in response to a painful surgical stimulus. In the case of isoflurane 1 MAC ≃ 1.17% ≃ 0.243 mM for an adult at normal body temperature (Mapleson, 1996). It should be noted that in combination with other anesthetic agents like nitrous oxide, less than 1 MAC of isoflurane will be required to reach the 50% end-point.

It is straightforward to introduce Equations (12, 13) to the Liley model by changing the Γ_*lk*_ according to isoflurane concentration, i.e., Γ_*lk*_ → Γ_*lk*_(*c*) = Γ^0^_*lk*_*H*_*l*_(*c*) with the Γ^0^_*lk*_ now having the same values as the Γ_*lk*_ had in the standard Liley model. However, Equations (9–11) are more problematic. The decay time of the alpha function in Equation (5) changes linearly with its rise time, thus one cannot match the experimental result that only the decay time is prolonged under anesthesia. Consequently, the following modification of Equation (2) was introduced (Bojak and Liley, 2005)

(14) $$\begin{array}{l} \left[\frac{1}{\gamma_{lk}(c)}\frac{\partial }{\partial t}+1\right]\left[\frac{1}{{\tilde{\gamma }}_{lk}(c)}\frac{\partial }{\partial t}+1\right]I_{lk}\left(\vec{x},t\right)\\ \;=\frac{e^{\gamma_{lk}(c)\delta_{lk}}\Gamma_{lk}(c)}{\gamma_{lk}(c)}A_{lk}\left(\vec{x},t\right)\;, \end{array}$$

(15) $$\gamma_{lk}(c)=\frac{\varepsilon_{lk}(c)}{e^{\varepsilon_{lk}(c)}-1}\frac{1}{\delta_{lk}}\;,\;{\tilde{\gamma }}_{lk}(c)=e^{\varepsilon_{lk}(c)}\gamma_{lk}(c)\;.$$

Notably, for ε_*lk*_ → 0 one finds that ${\tilde{\gamma }}_{lk}\to \gamma_{lk}$, and γ_*lk*_ → 1/δ_*lk*_ with a removable discontinuity. Defining these variables as continuous with the limit, the new Equation (13) then becomes identical with the old Equation (2) in this limit. The corresponding response to a single pre-synaptic spike *A*_*lk*_(*t*) = δ(*t*) now becomes a bi-exponential function

(16) $$I_{lk}(t)=\frac{e^{\gamma_{lk}\delta_{lk}}\Gamma_{lk}}{\gamma_{lk}}\beta_{lk}(t)\;,$$

(17) $$\beta_{lk}(t)=\gamma_{lk}{\tilde{\gamma }}_{lk}\frac{e^{-\gamma_{lk}t}-e^{-{\tilde{\gamma }}_{lk}t}}{{\tilde{\gamma }}_{lk}-\gamma_{lk}}\Theta (t)\;,$$

where we have suppressed the concentration dependence. Again the pre-factor in Equation (16) is proportional to the charge transferred, since β_*lk*_(*t*) is normed to one for integration over time. Note that now *I*_*lk*_ (*t* = δ_*lk*_) = Γ_*lk*_, which for ε_*lk*_ = 0 becomes the previous result since in this limit again δ_*lk*_ = 1/γ_*lk*_. More generally, for ε_*lk*_ → 0 we have β_*lk*_(*t*) → α_*lk*_(*t*) at all times and the alpha function is the “sharpest” response β_*lk*_(*t*) ≥ α_*lk*_(*t*). Clearly with this new form we can keep the rise time parameter δ_*lk*_ constant, while changing the ε_*lk*_ so as to achieve a desired decay time ζ_*lk*_. Given the changes imposed by isoflurane in Equations (9–11), one can solve for the appropriate ε_*lk*_ numerically in dependence on the concentration *c*. However, here we will use the excellent approximation formula presented in Liley et al. (2011), which can be written as^3^

(18) $$\begin{array}{l} \varepsilon_{lk}(c)≃e^{2.5466-1.3394\kappa_{lk}(c)}\sqrt{\kappa_{lk}(c)-1}+\left(e^{-1.2699\left[\kappa_{lk}(c)-1\right]}-1\right)\\ \;\cdot \left[\frac{1}{\kappa_{lk}^{2}(c)}+{\text{W}}_{-1}\left(\frac{e^{-0.23630/\kappa_{lk}^{2}(c)}}{1-3.1462\kappa_{lk}(c)}\right)\right]\;, \end{array}$$

where *W*_−1_ is the −1 branch of the Lambert-W function. Here we assume that κ_*ek*_ = 1, thus ε_*ek*_ = 0, and only the inhibitory decay time is affected.

Equations (1–4, 7–8) represent a system of eight coupled non-linear PDEs that define the standard Liley model. Changing the PSPs of Equation (2) to those of Equations (14, 15) defines the extended Liley model. It is therein understood that 1/δ_*lk*_ of the extended Liley model equals γ_*lk*_ of the standard one, so that for ε_*lk*_ → 0 both become identical. Finally, Equations (9–13) parameterize the effect of isoflurane on the extended Liley system. Here Equations (12, 13) can be used straightforwardly as determining Γ_*lk*_(*c*), but in order to use Equations (10–11) one additionally needs Equation (18) to translate them into changes of the γ_*lk*_(*c*) and ${\tilde{\gamma }}_{lk}(c)$ parameters. In Bojak and Liley (2005) extensive parameter searches were performed. All selected parameter sets gave rise to a plausible resting EEG power spectrum (‘1/*f*’ low frequency activity with an alpha peak in the 8–13 Hz range) under noise driving, retained a stable equilibrium point for increasing isoflurane concentration and hence remained in a quasi-linear dynamical regime, and showed the experimentally observed drop of the alpha peak to low frequencies for increasing isoflurane concentration. Some parameter sets furthermore exhibited a so-called “bi-phasic” transient surge in total power during simulated anesthesia induction, as observed in several experiments (Kuizenga et al., 1998, 2001). The parameter values used in this paper correspond to one of these “bi-phasic” parameter sets, and are listed in Table 1.

**Table 1:** **Mean population parameter values used to obtain bursting in the Liley model**.

**Definition**	**Excitatory (target)**		**Inhibitory (target)**	
Passive membrane decay times	τ_*e*_	65.815 ms	τ_*i*_	130.13 ms
Resting membrane potentials	*h*^r^_*e*%_	−78.422 mV	*h*^r^_*i*%_	−72.959 mV
Maximum firing rates	*S*^max^_*e*_	0.39535/ms	*S*^max^_*i*_	0.15439/ms
Firing thresholds (FTs)	μ_*e*_	−51.656 mV	μ_*i*_	−47.267 mV
Standard deviations of FTs	σ_*e*_	2.8669 mV	σ_*i*_	4.3250 mV
Synaptic recovery times	τ^rec^_*e*_	800.00 ms	τ^rec^_*i*_	600.00 ms
Synaptic depletion factor	*f*_*e*_	1.2500	*f*_*i*_	0.17500
**EXCITATORY SOURCE**				
Reversal potentials	*h*^eq^_*ee*_	−5.7891 mV	*h*^eq^_*ei*_	−1.6566 mV
PSP peak amplitudes	Γ_*ee*_	0.18424 mV	Γ_*ei*_	1.8771 mV
PSP rise times to peak	δ_*ee*_	9.1059 ms	δ_*ei*_	1.2103 ms
Number of intracortical synapses	*N*^β^_*ee*_	3410.8	*N*^β^_*ei*_	2738.9
Number of cortico-cortical synapses	*N*^α^_*ee*_	3616.3	*N*^α^_*ei*_	2905.1
Cortico-cortical decay scale	λ_*ee*_	24.000 mm	λ_*ei*_	24.000 mm
Cortico-cortical conduction velocity	*v*_*ee*_	2.1042 mm/ms	*v*_*ei*_	2.1042 mm/ms
Rate of extracortical input	*p*_*ee*_	9.3193/ms	*p*_*ei*_	3.1563/ms
**INHIBITORY SOURCE**				
Reversal potentials	*h*^eq^_*ie*_	−86.675 mV	*h*^eq^_*ii*_	−84.596 mV
PSP peak amplitudes	Γ_*ie*_	1.5969 mV	Γ_*ii*_	1.0838 mV
PSP rise times to peak	δ_*ie*_	2.5985 ms	δ_*ii*_	9.6946 ms
Number of intracortical synapses	*N*^β^_*ie*_	863.89	*N*^β^_*ii*_	267.92

### 2.2. Slow and activity-dependent synaptic bursting mechanism

In this work we consider receptor desensitization and synaptic vesicle depletion during periods of high neuronal population activity, and the homeostatic recovery of synaptic readiness during periods of low neuronal activity, as the slow mechanism that can modulate the excitability of cortical tissue. Such activity-dependence of synaptic efficacy has been observed during burst suppression induced with isoflurane (Kroeger and Amzica, 2007). In practice, we will modify the maximum PSP amplitudes Γ_*lk*_ that can be obtained, which directly depend on the available pre-synaptic amount and post-synaptic impact of neurotransmitter. Instead of considering these quantities as parameters as in the extended Liley model (where they act as control parameters that can be changed according to the concentration of an anesthetic agent), we now consider them as variables with their own slow dynamics coupled to the neural activity. Our ansatz is a common phenomenological model for activity-dependent synaptic depression (Bressloff, 2012). It represents a rate-based version of the model proposed by Tsodyks and Markram (1997), under the assumption that the processes responsible for the recovery of synaptic efficacy evolve on a time scale much slower than those associated with that of synaptic depletion (e.g., receptor desensitization and synaptic vesicle depletion):

(19) $$\frac{\partial \Gamma_{lk}\left(\vec{x},t\right)}{\partial t}=\frac{\Gamma_{lk}^{\text{r}}-\Gamma_{lk}\left(\vec{x},t\right)}{\tau_{l}^{\text{rec}}}-\rho_{l}^{\text{dep}}S_{l}\left[h_{l}\left(\vec{x},t\right)\right]\Gamma_{lk}\left(\vec{x},t\right)\;,$$

with *l*, *k* ∈ {*e*, *i*} indicating the excitatory and inhibitory subpopulations and *S*_*l*_(*h*_*l*_) is the local population firing rate of Equation (7), as before. Local neurotransmitter depletion is here considered to be directly proportional both to the strength of the PSPs, represented by Γ_*lk*_ itself, and to their frequency, represented by *S*_*l*_. In the absence of neural activity *S*_*l*_ = 0/*s*, there will be an exponential return of Γ_*lk*_ to the resting value Γ^r^_*lk*_ with a characteristic recovery time τ^rec^_*l*_. However, if there is no recovery τ^rec^_*l*_ → ∞ and we have constant neural activity *S*_*l*_ > 0/*s*, then Γ_*lk*_ will exponentially decay to zero with a characteristic depletion time 1/(ρ^dep^_*l*_ *S*_*l*_). Note that we have assumed that in the *pre-synaptic* recovery and decay there is no dependence on the target (on the index *k*), since these processes will be determined by the activity of the source. However, in the *post-synaptic* impact on the maximum amplitude of the PSP, we allow a dependence on the target, since the response will depend on the morphology and physiology of the receiving neurons.

Now consider the case where we have both depletion and recovery. We will choose some homogeneous *h*_*l*_($\vec{x}$, *t*) ≡ *h*^0^_*l*_ so that *S*_*l*_(*h*^0^_*l*_) > 0/*s*. The synaptic system will then converge everywhere to an equilibrium value easily calculated by setting the left hand side of Equation (19) to zero:

(20) $$\Gamma_{lk}\left(\vec{x},t\right)\;\to \;\Gamma_{lk}^{0}=\frac{\Gamma_{lk}^{\text{r}}}{1+\tau_{l}^{\text{rec}}\rho_{l}^{\text{dep}}S_{l}\left(h_{l}^{0}\right)}\equiv \frac{\Gamma_{lk}^{\text{r}}}{1+f_{l}}\;,$$

(21) $$\;f_{l}\equiv \tau_{l}^{\text{rec}}\rho_{l}^{\text{dep}}S_{l}(h_{l}^{0})=\frac{\Gamma_{lk}^{\text{r}}}{\Gamma_{lk}^{0}}-1\;.$$

This equilibrium value is always smaller than the resting one, i.e., *f*_*l*_ > 0. We can scale our ansatz in terms of this equilibrium value and then obtain

(22) $$\Gamma_{lk}\left(\vec{x},t\right)\equiv \Gamma_{lk}^{0}C_{l}\left(\vec{x},t\right)\;,$$

(23) $$\tau_{l}^{\text{rec}}\frac{\partial C_{l}\left(\vec{x},t\right)}{\partial t}=1+f_{l}-\left(1+\frac{S_{l}\left[h_{l}\left(\vec{x},t\right)\right]}{S_{l}\left(h_{l}^{0}\right)}f_{l}\right)C_{l}\left(\vec{x},t\right)\text{​}.\;$$

This scaling conveniently removes the post-synaptic dependence from the dynamical equations. Hence if we assume that the scaled initial conditions are identical Γ_*le*_($\vec{x}$, *t* = 0)/Γ^0^_*le*_ = Γ_*li*_($\vec{x}$, *t* = 0)/Γ^0^_*li*_, then in practice we only have to solve the two equations of Equation (23) instead of the four of Equation (19), obtaining the four potentially different peak amplitudes via the scaling in Equation (22). Our choice for the initial conditions is to start them all at equilibrium Γ_*lk*_($\vec{x}$, *t* = 0) = Γ^0^_*lk*_, thus *C*_*e*_($\vec{x}$, *t* = 0) = *C*_*i*_($\vec{x}$, *t* = 0) = 1, and then we can use these reduced computations with subsequent scaling. The only constraint we have imposed on the mean membrane potentials *h*^0^_*l*_ here is that they should lead to non-zero population firing rates, which however is always the case unless one assumes unphysiological infinite polarization. The depletion coupling constants between the neural activity and the peak amplitude ρ^dep^_*l*_ are empirically unknown, and would have to be determined laboriously from observations of dynamical changes of synaptic efficacy. However, in terms of the model proposed here, if one specifies the equilibrium values of the soma membrane potentials *h*^0^_*l*_ and how much the neurotransmitter reservoir is depleted at the corresponding activity levels (Γ^0^_*lk*_ vs. Γ^r^_*lk*_), then this in turn determines the depletion coupling constants

(24) $$\rho_{l}^{\text{dep}}=\frac{f_{l}}{\tau_{l}^{\text{rec}}S_{l}\left(h_{l}^{0}\right)}=\frac{\Gamma_{lk}^{\text{r}}/\Gamma_{lk}^{0}-1}{\tau_{l}^{\text{rec}}S_{l}\left(h_{l}^{0}\right)}\;.$$

In practice we make an implicit choice of the coupling constants by choosing the *f*_*l*_ for the system.

Now we wish to combine this synaptic system with the extended Liley model for anesthesia. For the parameter sets provided by Bojak and Liley (2005), that model has stable equilibrium points. That is to say, if we re-write the extended Liley model in the abstract form

(25) $$\vec{s}\left(\vec{x},t\right)\equiv {\left(h_{e},\;h_{i},\;I_{ee},\;I_{ei},\;I_{i.e.},\;I_{ii},\;\Phi_{ee},\;\Phi_{ei}\right)}^{T}\left(\vec{x},t\right)\;,$$

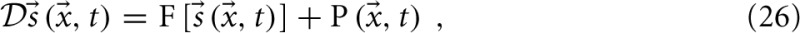

with a suitable differential operator 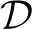, a function F and a noise drive P, then there exists a solution

(27) $$F[{\vec{s}}^{*}]=0\;,$$

so that for *P*($\vec{x}$, *t*) = 0 the system is static. Furthermore, since this equilibrium is stable, after small and transient disturbances the system will return dynamically to $\vec{s}$^*^. We now make the following replacements in the extended Liley system

(28) $$\Gamma_{lk}\;\to \;\Gamma_{lk}\left(\vec{x},t\right)\;,$$

i.e., we replace the parameter values Γ_*lk*_ of the extended Liley model with the variables Γ_*lk*_($\vec{x}$, *t*) of the synaptic system that we have just described. Together with the coupling to the neural activity explicit in Equation (23) this closes the combined system, which we will call the bursting Liley model henceforth. We now make the following convenient choices

(29) $$\vec{s}\left(\vec{x},t=0\right)={\vec{s}}^{*},\;C_{l}\left(\vec{x},t=0\right)=1,\;h_{l}^{0}=h_{l}^{*},\;\Gamma_{lk}^{0}=\Gamma_{lk},$$

This homogeneous initial state of the bursting Liley model must now be an equilibrium point by construction: while Equation (27) is calculated with the parameters Γ_*lk*_, we have arranged it so that the equilibrium value Γ^0^_*lk*_ of the synaptic system at the resulting mean soma membrane potentials has the same value as that parameter. This is simply achieved by fixing the Γ^r^_*lk*_ for a given *f*_*l*_ according to Equation (20), i.e., Γ^r^_*lk*_ = Γ^0^_*lk*_(1 + *f*_*l*_). Hence the equilibrium of one system is compatible with that of the other, and if we start them off in their respective equilibrium states nothing will change. However, there is no guarantee that this constructed equilibrium point of the bursting Liley model will be stable.

Previously, we had incorporated the effects of isoflurane into the extended Liley model in part by replacing the standard parameter Γ^0^_*lk*_ according to Equations (12, 13) with the anesthesia-dependent Γ_*lk*_(*c*) = Γ^0^_*lk*_ *H*_*l*_(*c*). In the bursting Liley model these parameters have become state variables with their own dynamics due to synaptic depletion and recovery. Hence the synaptic dynamics pertaining to the (pre-synaptic) source likewise must be multiplied by the anesthesia-dependent *H*_*l*_(*c*) to compute the (post-synaptic) amplitude induced at the target:

(30) $$\begin{array}{l} \Gamma_{lk}\left(\vec{x},t,c\right)\equiv \Gamma_{lk}\left(\vec{x},t\right)H_{l}(c)=\Gamma_{lk}^{0}C_{l}\left(\vec{x},t\right)H_{l}(c)\\ \;=\Gamma_{lk}(c)C_{l}\left(\vec{x},t\right). \end{array}$$

However, since the synaptic dynamics are now coupled to the spatially variable cortical activity, we need to adjust our synaptic inputs to Equation (14):

(31) $$\begin{array}{l} A_{ek}\left(\vec{x},t\right)=N_{ek}^{\beta }C_{e}\left(\vec{x},t\right)S_{e}\left[h_{e}\left(\vec{x},t\right)\right]+N_{ek}^{\alpha }\Phi_{ek}\left(\vec{x},t\right)\\ \;+p_{ek}\left(\vec{x},t\right)\text{​}, \end{array}$$

(32) $$A_{ik}\left(\vec{x},t\right)=N_{ik}^{\beta }C_{i}\left(\vec{x},t\right)S_{i}\left[h_{i}\left(\vec{x},t\right)\right],\text{​​​}$$

(33) $$\text{​}\left[\text{​}{\left(\text{​}\frac{1}{v_{ek}}\frac{\partial }{\partial t}+\frac{1}{\lambda_{ek}}\right)}^{2}-\nabla^{2}\text{​}\right]\text{​ }\Phi_{ek}\left(\vec{x},t\right)=\frac{1}{\lambda_{ek}^{2}}C_{e}\left(\vec{x},t\right)S_{e}\left[h_{e}\left(\vec{x},t\right)\right]\text{ ​}.$$

Here the first term in *A*_*lk*_ is multiplied with *C*_*l*_($\vec{x}$, *t*) at the same time and position, since it represents local and quasi-instantaneous synaptic input. For the second term of *A*_*ek*_, the *C*_*e*_($\vec{x}$, *t*) term is instead included through Equation (33). The right hand side of this propagation equation, while written in terms of the ($\vec{x}$, *t*), effectively encodes the signal at a distance location $\vec{x}$′, sampled there at time *t*′, and then transported with velocity *v*_*ek*_ to the local position $\vec{x}$ with a conduction delay *t* − *t*′; see for example (Bojak and Liley, 2010) for an explanation in terms of Green's functions. Thus, we now propagate the firing rate as scaled by the pre-synaptic efficacy of the neural populations at a distant position $\vec{x}$′ at the time *t*′. We note that this is not quite physiologically accurate either, since the synaptic dynamics should be evaluated at ($\vec{x}$, *t*), not ($\vec{x}$′, *t*′), albeit driven with the firing rates from $\vec{x}$′ delayed by *t* − *t*′. This could be achieved by setting *N*^β^_*ek*_*C*_*e*_ → *N*^β^_*ek*_*C*^*S*^_*e*_ and *N*^α^_*ek*_Φ_*ek*_ → *N*^α^_*ek*_ *C*^Φ^_*ek*_Φ_*ek*_ in Equation (31), removing *C*_*e*_ in Equation (33), and then tracking separately the spatiotemporal dynamics of *C*^*S*^_*e*_ and *C*^Φ^_*ek*_, respectively, where the latter would have Φ_*ek*_ instead of *S*_*e*_ in Equation (23). However, this would double the effort for computing the synaptic dynamics and could have potentially undesirable consequences for the separation of distant sources, see the Discussion for further detail. Finally, for simplicity we have assumed here that the extracortical input *p*_*ek*_ remains unchanged. This assumption will likely need to be improved upon for greater physiological realism, i.e., we do expect that in particular thalamic activity will also be modified by anesthesia. However, our current focus on only the cortical side allows us to highlight the proposed bursting mechanism without potential interference from complex interactions between extracortical and cortical structures. Given the assumption that *p*_*ek*_ is constant (or in the case of *p*_*ee*_, that its mean is constant), we would not expect the pre-synaptic efficacy to change. Overall, if we switch off the synaptic dynamics *C*_*l*_ ≡ 1, we recover exactly the extended Liley model of Bojak and Liley (2005).

The bursting Liley model hence consists of Equations (1, 7, 14, 15, 21–23, 30–33), with the influence of isoflurane anesthesia being parameterized by Equations (9–13, 18). In practice we choose the *f*_*l*_ > 0, and as before use the “combined fixed point” initial state of Equation (29). The parameter values we have chosen are listed in Table 1. For the synaptic system, we have followed qualitatively the work of Tsodyks and Markram (1997) in assuming a possible range of about 250 ms to 1000 ms for τ^rec^_*l*_, and values between 0.1 and 2.0 for *f*_*l*_. The values used in this paper were chosen after computational experimentation with various settings, and were selected because they lead to bursting only for relatively large concentrations of isoflurane. Clearly, more systematic and comprehensive scans of the available parameter space and better understanding of the dependence of the observed dynamics on these parameter values are needed in order to elucidate the mechanisms proposed here. However, it takes considerable computing time to simulate such large spatial systems. In order to accomplish a proper analysis of the parametric dependencies, one will likely need to find approximate but rapid evaluation methods, similar to replacing the full simulation with an eigenvalue calculation as in Bojak and Liley (2005). The development of such methods is beyond the scope of this article, here we want to demonstrate in a pilot study that we can qualitatively reproduce the spatial differentiation in burst suppression that has been observed experimentally.

### 2.3. Numerical simulations

All our simulations are performed on a two-dimensional cortical sheet discretized by a 512 × 512 numerical grid, where we assume a grid spacing of Δ*x* = Δ*y* = 1 mm. The effective simulation area of 2,621.44 cm^2^ corresponds roughly to the size of an entire human cortex (Im et al., 2008). Smaller grids, with or without larger grid spacing, have been used to investigate parameter dependencies more rapidly, but the results presented in this paper were all obtained on this standard grid. In order to avoid boundary effects we have made the numerical grid toroidal, i.e., if we number the grid points 0–511 along one dimension from left to right, then the grid point to the left of 0 is 511, and the grid point to the right of 511 is 0, and this is true for both dimensions. Obviously such a geometry is artificial as compared to the real brain. However, since it leaves all numerical grid points entirely equivalent, this together with the isotropic and homogeneous “background” connectivity implicit in the PDE propagation makes minimal assumptions about the actual geometry and specific connectivity of the brain. Basically, it represents a kind of anatomical “null hypothesis” from which any anatomical detail will deviate; and the more homogeneous the brain turns out to be in an effective sense, the better this approximation will represent its activity. As argued above, at significantly increased computational costs one can improve this description with neural mass meshes, but this is not expected to change the results obtained here at least qualitatively.

The only dependence on space is found in the propagation PDE of Equation (33). Hence the other dynamics are effectively described by a set of independent ordinary differential equations (ODEs) in time at every grid point. We solve all these ODEs with the following simple method: First, any higher time derivatives are turned into first derivatives by defining auxiliary variables, e.g., *d*^2^*g*/*dt*^2^ = *f*(*g*) becomes $dg/dt=\tilde{g}$ and $d\tilde{g}/dt=f(g)$. Next, we solve these first order ODE systems with the forward Euler method. Obviously many more efficient numerical schemes exist. But in our experience they occasionally fail for specific parameter settings with the Liley model, whereas the forward Euler method always remains stable. Thus, we trade speed for guaranteed stability here. In the propagation PDE, the Laplacian is approximated by a five point stencil, i.e., to estimate the Laplacian at a grid point, we use the value at that point and those of its four horizontal and vertical nearest grid neighbors. We find that numerical stability is increased, if in this PDE we likewise estimate the second derivative in time directly by considering the current, previous and future values (and solving for the future one), rather than first rewriting them into first order derivatives as for the other dynamics. We use MPI-C to parallelize the computation across multiple nodes (threads and/or cores). This involves splitting up the grid into patches assigned to the individual nodes. We note that since the only spatial dependence in the dynamics arises from the Laplacian, and since we approximate it with a five point stencil, the only required communication between these nodes is that of the proximate part of the one grid point deep boundary of the local patch to the nodes working on the adjacent patches. This limited need for communication between nodes allows for very efficient parallel computation.

How the noise driving the system is generated and filtered in a mathematical sense has been described above, here we will add the following technical comments: The temporal Catmull-Rom spline filter is obviously local in space, and hence in a parallel setting can be performed by every individual compute node on the grid points assigned to it. However, the initial noise generation is done in Fourier space, to allow spatial filtering by a simple multiplication at every (Fourier space) grid point, followed by an inverse Fourier transformation. We use FFTW (Frigo and Johnson, 2005) to perform the inverse Fourier transform in parallel across the available compute nodes. This means that the random number generation and the Fourier space filtering can be done local in each node on its part of the Fourier grid, while FFTW organizes the communications between the nodes involved in the inverse Fourier transform.

The time step used in our simulations is Δ*t* = 5 · 10^−5^ s, which for our chosen grid spacing is sufficient to achieve stable and convergent results. However, we save the simulation results neither at every time step, nor the entire system state, nor at the internal double floating point precision (8 Bytes). The reason is that the 10 state variables of the bursting Liley model saved for 512 × 512 grid points at 8 bytes per point already would require 20 MB of hard disk space per time step, and thus at full time resolution a mere 3 s of run time would generate more than 1 TB of data. In practice, we typically save between one and four selected state variables with 250 Hz, converting to single floating point precision (4 Bytes) in the output. This still generates data files of many GB for our longest runs. Finally, even this data reduction is not sufficient to generate suitably sized animations of our results. Basically, the fine detail of a 512 × 512 grid leads to low compression efficiency of the employed (H. 264) movie codec. For producing animations we hence tile the output grid into squares of 4 × 4 grid points and average over these to obtain an effective 128 × 128 grid with smoother values that compress better. This explains the mild visual disparity between our figures (at full 512 × 512 resolution) and the animations, even though they are produced from the same underlying data set. We also produce video frames at an even lower sampling rate in time, and we use variable sampling rates to selectively speed up uneventful parts of the video. A time counter in the videos keeps track of the sampling rate, and occasional choppiness and blurriness in the videos does not reflect actual discontinuities in the simulations but merely low sampling rates and aggressive video compression.

## 3. Results

**Figure 2:**
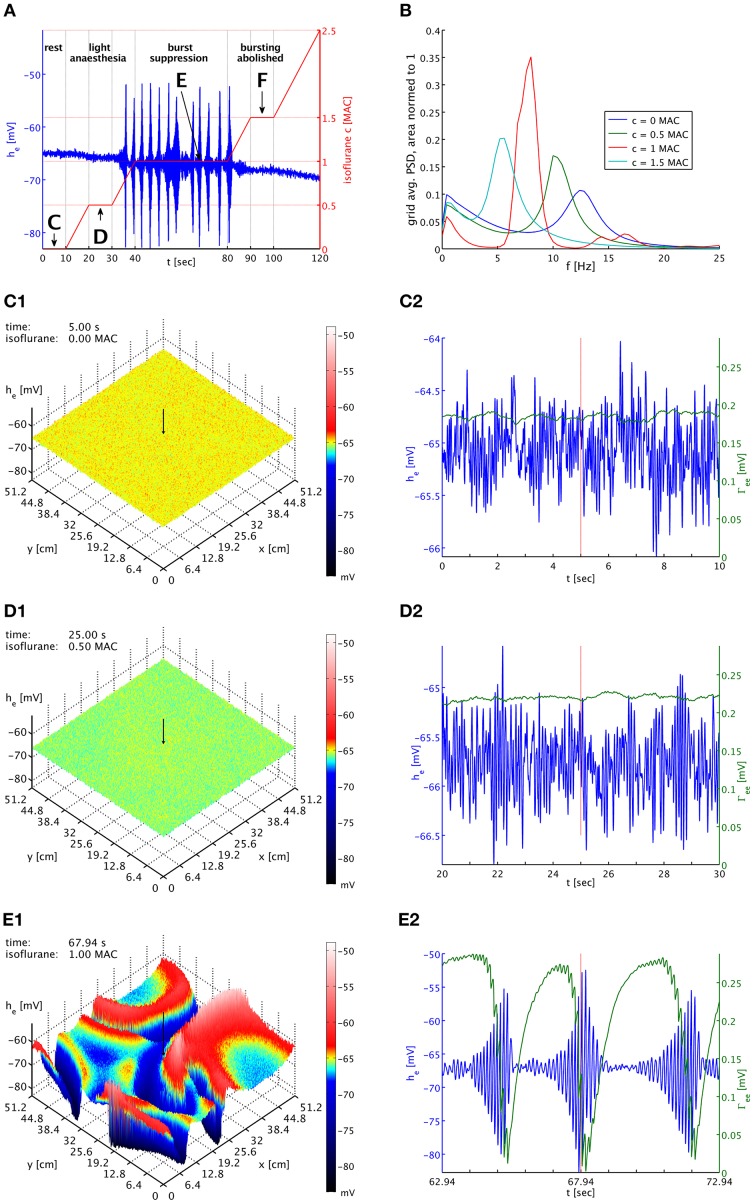
**(A)** Imposed concentration of isoflurane (red curve), and the *h*_*e*_ response (blue curve) at the cortical location indicated by black arrows in the snapshot panels below. Different plateaus of concentration are labeled “C,” “D,” “E,” and “F.” Arrows point to the central times of the corresponding time series shown below. **(B)** PSDs of *h*_*e*_ averaged over the entire grid and normed to unit area for plateaus “C” (blue), “D” (green), “E” (red), and “F” (cyan). The motion of the alpha peak to lower frequencies persists qualitatively into the burst suppression phase “E” at much increased power. **(C1)** Snapshot of the *h*_*e*_ activity of the cortical surface at 0 MAC isoflurane. The size of *h*_*e*_ is indicated by both height and color, cf. the color bar. A black arrow shows the position from which the corresponding time series were recorded. **(C2)** Time series of *h*_*e*_ (blue) and Γ_*ee*_ (green) over the 10 s of the “C” plateau. Regular alpha rhythms in *h*_*e*_ and slow Γ_*ee*_ oscillations around the standard value Γ^0^_*ee*_ can be seen. **(D1)** Snapshot at 0.5 MAC. **(D2)** Time series of the “D” plateau. The oscillations of *h*_*e*_ have larger amplitude at a lower average. The slow Γ_*ee*_ oscillations now occur at an elevated level. **(E1)** Snapshot at 1 MAC. Burst suppression patterns have emerged and move across the cortical surface. **(E2)** Time series of the “E” plateau. Burst suppression is apparent both in *h*_*e*_ and Γ_*ee*_, with a rapid drop in Γ_*ee*_ caused by the strongest *h*_*e*_ oscillations. An animation of this simulation is provided as Movie 1 in the Supplementary Material.

We explore the influence of isoflurane on the model in a long simulation run presented in Figure 2. The entire simulation also has been animated as Movie 1, included in the Supplementary Material. In Figure 2A we show the time course of the isoflurane concentration that we have imposed. First the system is run free of anesthesia (0 MAC) for 10 s. We call this the first plateau in the following. The equilibrium values of the system are used as initial conditions. Hence there are no transient dynamics, which allows us to estimate a power spectral density (PSD) from the *h*_*e*_ time series. Then we increase the concentration linearly to 0.5 MAC (equivalent to 0.1215 mM or 0.585% inspired at normal body temperature) over 10 s, and keep the system at this concentration for another 10 s. This second plateau corresponds to a light anesthesia state, without burst suppression, and again we can estimate a PSD here. Next we increase the concentration linearly to 1.0 MAC (equivalent to 0.243 mM or 1.17% inspired), and keep the system there for 40 s. This third plateau corresponds to a state of deep anesthesia, with burst suppression, and we can estimate a PSD here as well. After that, we increase the isoflurane concentration again for 10 s to 1.5 MAC (equivalent to 0.3645 mM or 1.755% inspired), and maintain it at this value for 10 s. Bursting is abolished at this fourth plateau, and we compute another PSD here. Finally, we raise the concentration for another 20 s up to 2.5 MAC (equivalent to 0.6075 mM or 2.925% inspired). This demonstrates that the system has finally returned to a regime without bursting.

In Figure 2B, we see PSDs estimated over these plateaus. At each concentration, we have calculated PSDs for every individual grid point from the *h*_*e*_ time series for the entire duration of the plateau, using a Welch estimate with a 2.5 s window and 50% overlap, and then have averaged the resulting 262,144 PSDs. We have normed these average PSDs to have unit area, i.e., their total power over all frequencies is one. This makes it easier to compare them visually. Please note that the parameter set used here is a “bi-phasic” one, see the discussion in Bojak and Liley (2005). Consequently, the total power at the second plateau is actually increased over that at the first plateau by a factor of 1.26. We see the characteristic shift of the alpha resonance to lower frequencies, in this case initially accompanied by a sharpening of the peak. Without the slow synaptic system as in Bojak and Liley (2005), a further increase of the isoflurane concentration would move the former alpha peak to ever lower frequencies, accompanied eventually by strong damping of the peak and a reduction of the total power, completing the bi-phasic power change. However, with the introduction of the slow synaptic system we see a burst suppression pattern emerge, and the large amplitude oscillations imply a drastic increase in total power by a factor 131 over the resting values. Yet we see that the PSD obtained from the third plateau—as far as frequency content is concerned—roughly follows what is expected without the synaptic system: The majority of the power, which is generated by the large bursts, is located where one would expect to see the former alpha resonance in the previous model of Bojak and Liley (2005). In other words, the bursts roughly conserve the regular dynamics of the system, in particular of the former alpha resonance. At the fourth plateau the system has ceased to burst but still shows elevated total power 1.44 times larger than at rest.

In Figure 2C1 we see the system at rest under noise drive. The visible structure is hence basically that of the spatial correlations we have included in the noise. The corresponding time series in Figure 2C2 shows the typical waxing and waning of a resting alpha rhythm in *h*_*e*_. Unsurprisingly, Γ_*ee*_ oscillates slowly at values about the equilibrium value of the slow synaptic system Γ^0^_*ee*_, cf. Table 1. In Figures 2D1,D2 we see the corresponding state at light anesthesia. The overall *h*_*e*_ is now lower across the grid, indicating smaller firing rates on average. However, as we can see in the time series the amplitude of the oscillations has increased, corresponding to the power increase expected for this “bi-phasic” parameter set. The oscillation frequency also has become lower, though this is easier to see in the PSDs of Figure 2B. We see that Γ_*ee*_ is still oscillating slowly, but around values somewhat higher than Γ^0^_*ee*_, because the synaptic resources are not as rapidly depleted by the reduced excitatory firing rate of the depressed *h*_*e*_.

In Figure 2E1 burst suppression patterns have emerged. These patterns are clearly independent of the noise drive. In the Movie 1, included in the Supplementary Material, one can see how this is a snapshot of “burst waves” moving across cortex, with centers of burst activity spontaneously forming and disappearing. The geometry of these excitations is complex and constantly changing. We see in Figure 2E2 that the strongest oscillations in *h*_*e*_ are associated with a rapid drop in Γ_*ee*_ due to the synaptic depletion during these periods of high firing. This lowering of Γ_*ee*_ quickly suppresses the burst by reducing the self-excitation of cortex. This is then followed by a recovery to values of Γ_*ee*_ that are large compared to those at rest or light anesthesia. This recovery to high values of Γ_*ee*_ is driven by the isoflurane-induced reduction in the mean excitatory firing rate during the suppressed periods. In turn, these strong PSPs eventually destabilize the neural system, leading to another burst. The burst suppression pattern persists as the anesthesia concentration is being increased again, up to quite high concentrations. In Movie 1, one can see that the amplitudes of the “burst waves” eventually become smaller and smaller until the burst activity fades away into regular noise driven activity. We do not show here corresponding plots for the fourth plateau with abolished bursting, but they would look similar to Figures 2D1,D2 with a further reduced mean value *h*_*e*_, even lower oscillation frequency, and a Γ_*ee*_ that is on average even higher.

**Figure 3:**
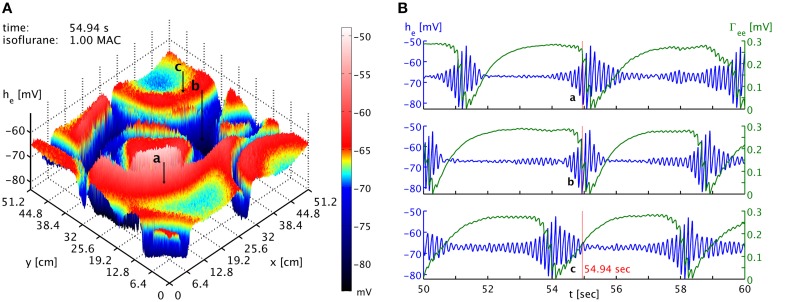
**(A)** Snapshot of the *h*_*e*_ activity of the cortical surface at simulation time 54.94 s under the influence of 1 MAC isoflurane. Black arrows with labels “a,” “b,” and “c” point to the cortical locations of the time series shown in the other panel. Note the toroidal boundaries, e.g., the circular burst front that appears cut off around (x,y) = (44.8, 0) cm continues at *x* = (44.8, 51.2) cm. **(B)** Time series of *h*_*e*_ (blue) and Γ_*ee*_ (green) taken from the three different positions marked as “a,” “b,” and “c” in the other panel. It is obvious that bursts are not generally synchronized in time at the different positions. Spatiotemporal correlations from propagating “burst waves” can occur, but are removed at larger distances by the interference from other emergent patterns. An animation of this simulation is provided as Movie 1 in the Supplementary Material.

The burst suppression phase shown in Movie 1 of the Supplementary Material makes obvious that one cannot expect global synchrony of the burst suppression across cortex. A multitude of transient spatiotemporal patterns emerge, travel across cortex, and dissolve. This is also shown in Figure 3, which shows time series from three well-separated locations on the simulated cortex at a specific point in time. There is little evidence of strong systematic correlations. While one might expect that the propagation of “burst waves” should lead to correlations with temporal delay at these distances, other burst features emerge across these spatial scales and interfere with the burst timing. Without observing spatiotemporal pattern globally, it hence will be difficult to find systematic correlations of the bursts at large distances. However, locally it may be possible to track the regular motion of burst patterns, e.g., at a point close to the one labeled “a” one might see bursting appear with a delay, characteristic for the “burst wave” passing through these two points sequentially. Overall, we expect stronger synchronization—or at least consistent phase differences from traveling patterns—at shorter distances, whereas at longer distances such correlations will be basically accidental. Thus, one would expect to see considerable spatial differentiation if one records from several spatial locations, as in Lewis et al. (2013). How many electrodes would be seen to burst at the same time would depend on the size and motion of the emerging spatiotemporal burst patterns.

Local variation of cortical tissue properties, reflected in the model evaluation by a change in the parameters, may also affect the ability of some part of cortex to participate (fully) in the spatiotemporal burst suppression dynamics. Such variation of tissue properties can be natural and develop intrinsically, or could be induced extrinsically by physical insult or the application of drugs. We have seen that bursts are associated with slow but large oscillations in the excitatory peak amplitudes of the PSPs. It is important to note that there are two different effects determining the general size of the Γ_*ek*_. On one hand, anesthesia is reducing Γ_*ek*_ directly as parameterized by the Hill factor *H*_*e*_(*c*), cf. Equation (30). On the other hand, the reduction in the average *h*_*e*_, mostly due to the strong prolongation of the inhibitory PSPs with anesthesia, means that the average excitatory firing rate *S*_*e*_ decreases. This in turn leads to less synaptic depletion and hence actually a rise in Γ_*ek*_, cf. Equation (19). The net effect with increasing concentration is actually an increase of Γ_*ek*_, and this is crucial for the onset of bursting. If one increases anesthesia further, eventually the Hill factor begins to dominate and Γ_*ek*_ decreases again.

The same can be said for the Γ_*ik*_, and the corresponding balance between the Hill factor *H*_*i*_(*c*) and the reduction in *S*_*i*_ for increasing anesthesia. However, we see that in the standard parameters the excitatory synaptic depletion factor *f*_*e*_ = 1.25 is much larger than the inhibitory one *f*_*i*_ = 0.175. This means that there is much less room for Γ_*ik*_ to grow, since the steady maximum is Γ^r^_*lk*_(*c*) = Γ^0^_*lk*_(1 + *f*_*l*_)*H*_*l*_(*c*). One simple idea for reducing the ability of cortical tissue to participate in burst suppression is hence to increase the growth of inhibition with anesthesia by raising *f*_*i*_. What do we expect to be the effect of this increased inhibition, in particular concerning the excitatory Γ_*ek*_? In general we expect *h*_*e*_ and *h*_*i*_ to decrease even more rapidly with increasing concentration of anesthesia, due to the boosted inhibition. But silencing the cortex also decreases synaptic depletion, so we actually expect a stronger initial Γ_*ek*_ growth for increasing anesthesia. It is hence not a priori clear whether the more rapid decrease in *h*_*e*_ or the more rapid increase in Γ_*ek*_ dominates, and in consequence whether bursting is abolished or maintained, respectively. We note that for our regular parameter set bursting is abolished at higher concentrations even though Γ_*ek*_ is still increasing, because *h*_*e*_ has then decreased too much. We may hence expect that an increase of *f*_*i*_ alone can stop the bursting.

**Figure 4:**
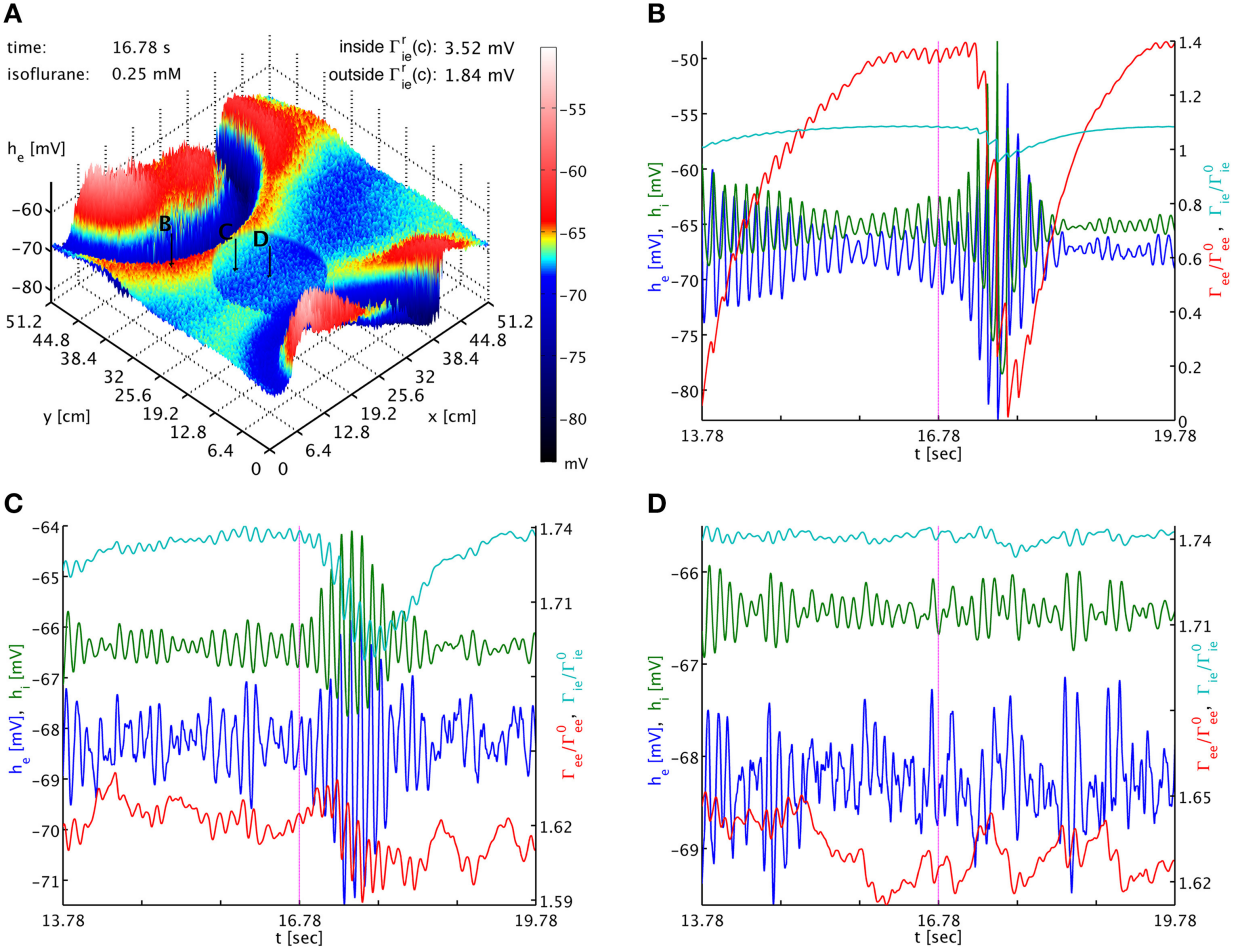
**(A)** Snapshot of the *h*_*e*_ activity of the cortical surface at simulation time 16.78 s under the influence of 0.25 mM isoflurane, where in a circular patch (center (*x*, *y*) = (20.0, 20.0) cm, radius 9.6 cm) the inhibitory synaptic depletion factor *f*_*i*_ has been increased from 0.175 to 1.25, leading to an increase of Γ^r^_*ie*,*ii*_ there by a factor 1.91. The size of *h*_*e*_ is indicated by both height and color, cf. the color bar. One can see that the circular patch does not participate in the burst suppression pattern. Black arrows with labels “B,” “C,” and “D” point to the cortical locations used in the other panels. **(B)** Six seconds long time series of *h*_*e*_ (blue), *h*_*i*_ (green), Γ_*ee*_/Γ^0^_*ee*_ (red), and Γ_*ie*_/Γ^0^_*ie*_ (cyan) around the time of the snapshot from a point outside of the circular patch. Burst suppression is clearly visible in all variables. **(C)** Time series from just inside the circular patch. There is no local burst suppression, but some of the outside burst activity spills in. **(D)** Time series taken from the center of the circular patch. There is neither local burst suppression nor spill-in. Animations of this simulation are provided as Movie 2 in the Supplementary Material.

To test this, we use a typical simulation at 0.25 mM isoflurane. However, in a circular patch of tissue we set *f*_*i*_ = 1.25 instead of the standard *f*_*i*_ = 0.175, while leaving this parameter at *f*_*i*_ = 0.175 across the rest of the cortical sheet. Using Γ^r^_*lk*_(*c*) = Γ^0^_*lk*_(1 + *f*_*l*_)*H*_*l*_(*c*), we have inside this patch Γ^r^_*ie*,*ii*_(0.25) = (3.5174, 2.3872) mV and outside Γ^r^_*ie*,*ii*_(0.25) = (1.8369, 1.2467) mV, respectively, while everywhere Γ^r^_*ee*,*ei*_(0.25) = (0.37703, 3.8414) mV. As shown in Figure 4, outside of the circular patch burst suppression patterns emerge as usual, see Figures 4A,B, while in the dead center of the circular patch there is no sign of such activity, see Figures 4A,D. Hence inside the patch the greater decrease in *h*_*e*_ was more effective than the greater increase in Γ_*ek*_, compare Figures 4B,D. Interestingly, at the border of the circular patch, see Figures 4A,C, we see largely the same state as for the center, but there appear to be some “quasi-bursts”. Actually, this is activity spilling into the circular patch from the outside through the propagation with Equation (33). The characteristic spatial decay scale of this propagation is λ_*ek*_ = 2.4 cm. Given a radius of 9.6 cm of the circular patch, we expect a signal from the outside to have fallen to less than 2% of its original value at the center. So it is unsurprising that any outside influence on the center is not obvious to the eye, but that close to the rim we see stronger echoes of the surrounding burst activity. In Movie 2 in the Supplementary Material *h*_*e*_ (top panel) and Γ_*ee*_ (bottom panel) animations are shown. Here one can observe the bursting waves collide with the circular patch, and then fade as they penetrate deeper. We note that an increase to for example *f*_*i*_ = 0.5 in the patch is not sufficient to abolish bursting in this manner, illustrating that it is the balance between the decrease in *h*_*e*_ and the increase in Γ_*ee*_ which determines whether self-excitation is possible.

**Figure 5:**
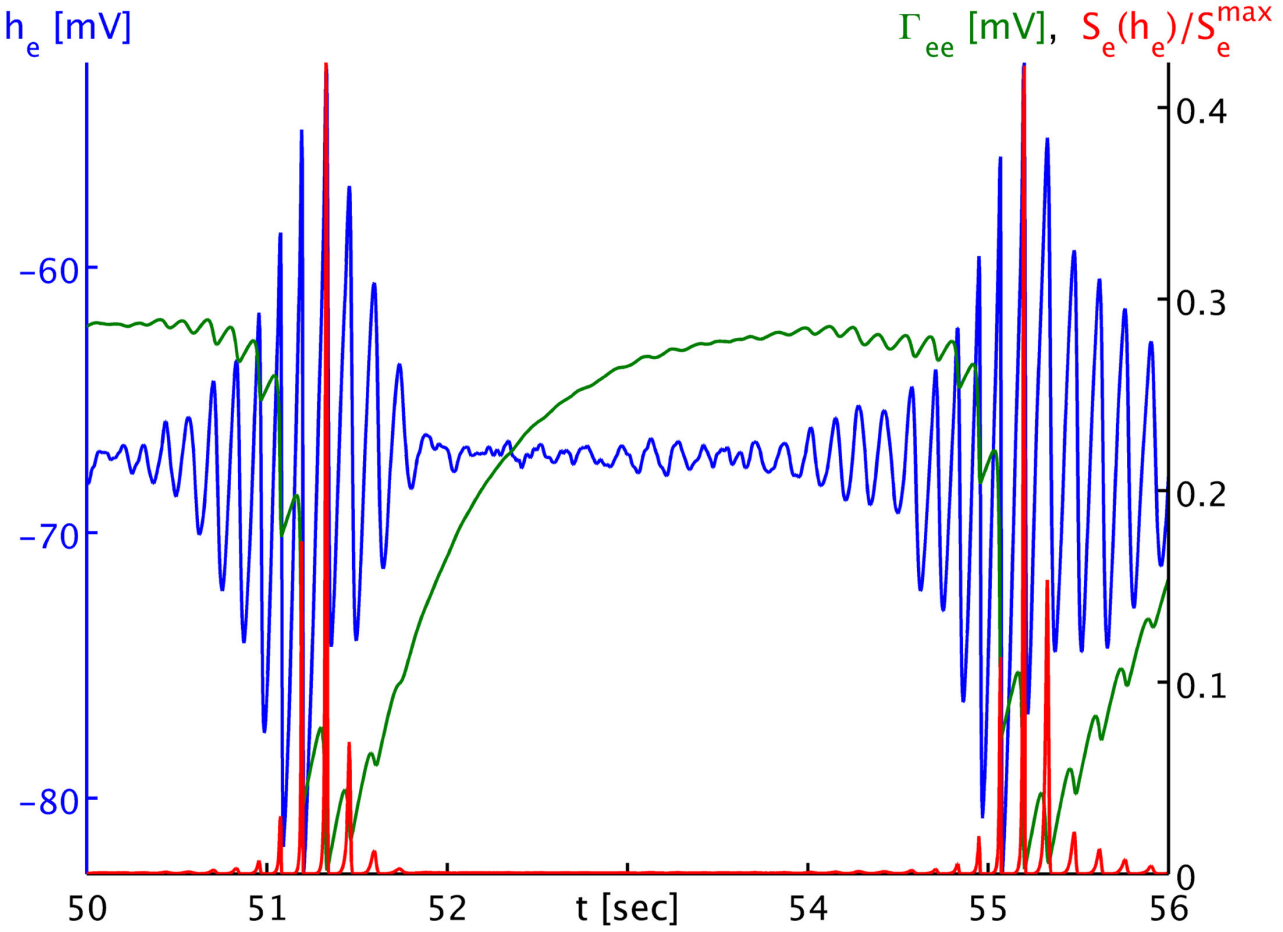
**Time series of the mean excitatory soma membrane potential *h*_*e*_ (blue), the excitatory post-synaptic peak amplitude Γ_*ee*_ (green) and the average excitatory firing rate normed to the maximum attainable rate *S*_*e*_(*h*_*e*_)/*S*^max^_*e*_ (red)**. Note that both Γ_*ee*_ and *S*_*e*_(*h*_*e*_)/*S*^max^_*e*_ map by value to the black ordinate on the right, though with different units. The time series shown here is part of the times series labeled “a” in Figure 3B. One sees that the strongly non-linear relationship between *h*_*e*_ and *S*_*e*_ in the anesthetic regime transforms the “symmetric” *h*_*e*_ oscillation that would be visible in local field potentials and the EEG during the burst phase into strong “spikes” in the firing rate *S*_*e*_, and consequently to a “jagged” appearance of the synaptic depletion of Γ_*ee*_.

The dynamics of Γ_*ee*_ are of course much slower than those of *h*_*e*_, and we can track the *h*_*e*_ burst fronts by the progression of the lowest dips and valleys in Γ_*ee*_. This corresponds to rapid synaptic depletion in high firing regions. The extent of “spill-in” from the outside into the circular patch is also easier to discern in Γ_*ee*_: the center of the patch remains at roughly constant values, while at the rim Γ_*ee*_ drops when high firing propagates into the patch. We note that the strong oscillations that one can see as Γ_*ee*_ drops rapidly in Figure 4C show up in the movie as a kind of “bouncing” rather than a smooth advance of the burst fronts. To understand this better, we provide Figure 5. It shows part of the time series labeled “a” in Figure 2B. However, in addition to *h*_*e*_ (blue curve) and Γ_*ee*_ (green curve), it also shows the mean excitatory firing rate *S*_*e*_(*h*_*e*_) as red curve. So that one ordinate can be used for both Γ_*ee*_ and *S*_*e*_(*h*_*e*_), we have normalized the latter by the maximum excitatory firing rate *S*_*e*_(*h*_*e*_)/*S*^max^_*e*_. The basically symmetric oscillations of the mean membrane potential *h*_*e*_ around an average value translate into strong “spikes” in the mean firing rate *S*_*e*_(*h*_*e*_). This is due to the sigmoidal nature of Equation (7), combined with the fact that the average *h*_*e*_ is about 5.5 standard deviations σ_*e*_ = 2.8669 mV below the average firing threshold μ_*e*_ = −51.656 mV, leading to low firing rates. Only the strongest depolarizations in the burst come close to this threshold—though even they do not quite reach it here, as we can see, since *S*_*e*_(μ_*e*_)/*S*^max^_*e*_ = 0.5 by definition. Thus, the relationship between *h*_*e*_ (local field potentials and EEG) with firing rates is highly non-linear in the anesthetic regime. It is obvious from Figure 5 that the jagged drop of Γ_*ee*_ is simply caused by strong synaptic depletion induced by “spikes” in the mean excitatory firing rate.

**Figure 6:**
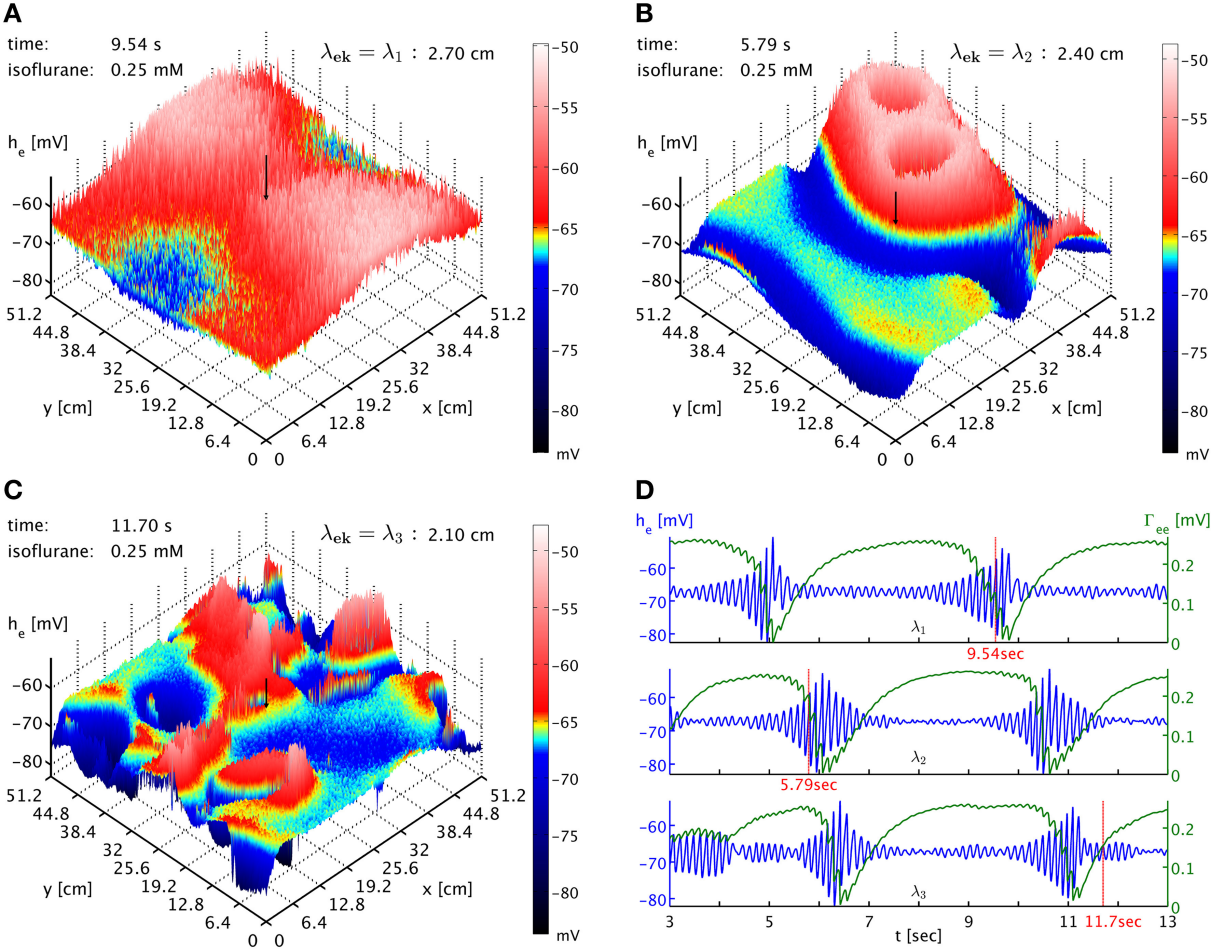
**(A)** Snapshot of the *h*_*e*_ activity of cortex at simulation time 9.54 s at 0.25 mM isoflurane with λ_*ek*_ = λ_1_ = 2.7 cm. A black arrow shows the cortical location at which the corresponding time was recorded. An animation is provided as Movie 3 in the Supplementary Material **(B)** Snapshot at simulation time 5.79 s with λ_*ek*_ = λ_2_ = 2.4 cm, the standard value. An animation is provided as Movie 4 in the Supplementary Material. The characteristic size of the burst patterns is reduced. **(C)** Snapshot at simulation time 11.70 s with λ_*ek*_ = λ_3_ = 2.1 cm. An animation is provided as Movie 5 in the Supplementary Material. The characteristic size of the burst patterns is reduced even further. **(D)** Time series of *h*_*e*_ (blue) and Γ_*ee*_ (green) taken from these three simulations, marked as “λ_1_,” “λ_2_,” and “λ_3_.” We see that inter-burst interval remains roughly the same.

Finally, we also considered the influence of the spatial scale of brain connectivity on the spatiotemporal expression of burst suppression. As mentioned above, in this simplified model it is represented by the parameter λ_*ek*_, the characteristic length scale of the exponential decay of activity propagated with Equation (33). Its regular value according to Table 1 is λ_*ek*_ = λ_2_ = 2.4 cm. This is a length scale one might associate with a brain region and cortico-cortical connections, in particular since the influence of activity at a point would be felt across a distance of several λ_*ek*_. We vary this length scale up λ_*ek*_ = λ_1_ = 2.7 cm and down λ_*ek*_ = λ_3_ = 2.1 cm to investigate the impact of brain connectivity on the dynamics. In Figure 6 we see the dependence of the spatiotemporal activity on adjusting this parameter. The spatial extent of the emerging burst patterns clearly becomes smaller as λ_*ek*_ is being decreased, cf. Figures 6A–C. This is particularly obvious in the corresponding animations for the different λ_*ek*_ values, Movies 3–5 of the Supplementary Material, where we can see that at λ_1_ large parts of cortex are recruited in the bursts, whereas for λ_3_ bursting is much more localized. Our standard λ_2_ represents an intermediate case. However, the time interval between bursts for these different λ_*ek*_ appears at first sight comparable, see Figure 6D. To be more quantitative, one can use the point where Γ_*ee*_ drops lowest as a convenient marker for the time of a burst peak. To carry out automatic computations for 50 s time series for every grid point, we select the deepest minimum within a specific continuous “burst peak region” defined by Γ_*ee*_ ≤ 0.05 as burst peak time, and we remove inter-burst intervals with Δ*t*_IBI_ < 1.0 s as not representative for single burst behavior. This cut removes “double-dipping” below our Γ_*ee*_ threshold, caused for example by two subsequent activity “spikes” in the same burst with just enough recovery in between to get above threshold (very small Δ*t*_IBI_) or the interference of two burst waves (small Δ*t*_IBI_). We find the following grid averages: 〈Δ*t*_IBI_〉_λ_1__ = (4.44 ± 0.26) s, 〈Δ*t*_IBI_〉_λ_2__ = (4.0 ± 1.1) s, and 〈Δ*t*_IBI_〉_λ_3__ = (3.5 ± 1.5) s. We see that the mean of Δ*t*_IBI_ is decreasing roughly by 0.5 s per 3 mm reduction of λ_*ek*_; whereas the standard deviation increases considerably with decreasing λ_*ek*_, reflecting the more diverse spatial distribution of the burst patterns. According to these simulations, we can expect that spatial differentiation—the size of the burst suppression patterns and their timing—is intimately linked to the effective extent of the brain connectivity propagating the burst activity.

## 4. Discussion

We find that the simulation of isoflurane induction with the model proposed here reproduces at least qualitatively the electrophysiological response that one can measure in the EEG (Foster et al., 2008). At light anesthesia there is an oscillatory shift to lower frequencies with higher amplitudes, then in deep anesthesia we find burst suppression patterns, and finally for even higher concentrations these bursts are abolished as cortex slowly heads toward electrotonic death. Significantly, in the burst suppression phase the prior regular activity of cortex is roughly “echoed” in the frequency content of the burst (Kroeger and Amzica, 2007; Ching et al., 2012), though of course the amplitude of the oscillations is much increased. We find that burst suppression results in dynamic and complex burst patterns that travel across cortex in waves, rather than remaining statically in place. The burst suppression phase is foreshadowed by the continuous elevation of peak PSP amplitudes, until finally these strong inputs destabilize the neural system into bursting. At maximum oscillation of the mean soma membrane of the neural population, strong depletion of the synaptic system leads to a sudden drop of the PSP peak amplitude, which suppresses the burst until the synaptic system is able to recover again. The relatively slow time scale of this recovery is what governs the periodicity of the bursts in the burst suppression regime in this model.

While we are mostly interested here in investigating the spatial differentiation of burst suppression qualitatively, the emergence of burst suppression in our simulations is also in rough quantitative agreement with what has been observed clinically. Because the emergence of burst suppression in the EEG represents a distinct endpoint, it has been proposed that it may be a suitable measure by which to titrate the administration of anesthesia to ensure optimal hypnosis. On this basis a variety of efforts have been made to estimate the concentration dependent emergence of burst suppression during anesthesia. It has been found that during sole agent isoflurane anesthesia, the burst suppression pattern can emerge at end-tidal concentrations as low as 1.2% (Hoffman and Edelman, 1995; Pilge et al., 2014), i.e., aqueous concentrations of ≃ 0.25 mM at 37°C. However when arterial blood concentrations of isoflurane have been measured the onset of burst suppression has been reported for levels as low as 34.9 μg/ml or ≃ 0.19 mM (Loomis et al., 1986) – close to the value at which we observed the onset of burst suppression in our model.

However, more importantly our model predicts the appearance of large-scale spatial burst patterns across cortex, which emerge, travel and disappear over time. In consequence, coherence of burst timing is mostly local, though one can expect to see characteristic burst onset time shifts in the case of burst patterns traveling across neighboring recording sites. Some of the simulated patterns become large in size intermittently, recruiting large parts of cortex and thus leading to more “global” correlations of burst timing. However, for the most part the complex spatiotemporal dynamics will lead to “local” correlations, with synchronization over large distances being mostly accidental. These predictions are at least qualitatively in line with the experimental observations of Lewis et al. (2013), see in particular their Figures 3, 4. We suggest that spatially dense experimental recordings may allow one to track such spatiotemporal burst patterns in detail. At least in principle it should be possible to reconstruct the underlying cortical state in terms of our model from such recordings, in particular if one tracks the activity over some length of time. The relatively slow spatiotemporal dynamics of the bursts may help in this regard.

We have shown here as well that increasing the inhibitory depletion factor *f*_*i*_, or equivalently the resting values Γ^*r*^_*ie*_ and Γ^*r*^_*ii*_ of the inhibitory peak amplitudes, can abolish bursting in the model in a localized manner (in a patch of simulated cortical tissue). This is due to affecting the balance of two competing effects: the decrease of *h*_*e*_ reduces, whereas the increase of Γ_*ee*_ increases, the capacity of cortex for self-excitation. For large enough increases of inhibition the former dominates the latter. This provides further possibilities for spatial differentiation in cortex as some tissue may have naturally higher inhibitory peak amplitude resting values, and hence be less capable of participating in burst suppression. In addition, it is expected that spatial heterogeneity in the cortical actions of anesthetics will contribute to the spatial differentiation of burst suppression. Most anesthetics that induce burst suppression are GABAergic agents, which in addition to enhancing inhibitory PSP action also produce increases in tonic inhibition, as well as reductions in tonic excitation by altering the activity of a variety of membrane bound channels that include two pore potassium channels, extrasynaptic GABA_A_ and nicotinic acetylcholinergic ionotropic receptors. However, these synaptic and extra-synaptic channels, which exist in multiple isoforms that are variably affected by the same anesthetic agent, are not distributed uniformly throughout cortex. Thus, we would expect no two regions of cortex to share exactly the same propensity to burst for a given anesthetic level. This is not reflected in the current work, but left for future studies.

The hypothesized synaptic basis for spatially heterogeneous burst suppression suggests that if one can accordingly manipulate the cortical tissue, then one can artificially suppress, abolish or even enhance its participation in bursts. Importantly, our model predicts that drugs increasing inhibition could have the paradoxical effect of increasing burst activity, depending on the precise balance of the *h*_*e*_ decrease and Γ_*ee*_ increase that they induce. In particular, one would typically expect that any paradoxical effects would occur at lower doses, since strongly increasing inhibition should eventually see the *h*_*e*_ decrease win over the Γ_*ee*_ increase. It is interesting to note that a wide variety of GABA_A_ modulators appear to have paradoxical effects at low doses, see for example (Bäckström et al., 2011) and references therein. Furthermore, our spatial model predicts that bursting activity of surrounding tissue can propagate into tissue that is incapable of bursting itself, to a depth depending on the density of synaptic connectivity, and lead there to “quasi-bursts” which simply reflect the dramatic variation of the synaptic input. However, if bursts are abolished by the mechanism suggested here, namely an increase in the inhibitory synaptic depletion factor *f*_*i*_, then this tissue would show particularly large inhibitory PSP amplitudes and largely constant (rather than strongly varying) excitatory ones. This suggests that one could experimentally distinguish between bursting and “quasi-bursting” tissue by monitoring the size of the PSPs.

Furthermore, we have shown that both the spatial extent of the burst patterns, and the timing of the bursts (in particular the interval between bursts) depend on the characteristic scale of the brain connectivity effectively involved in propagating this activity: the shorter range these connections, the smaller the regions of coherent burst activity, and the more rapidly one burst follows on after the other. While at present this constitutes a qualitative finding, and while the implementation of brain connectivity in our model (homogeneous, isotropic and exponentially reducing with distance) is too simplistic to speak directly to the complexity of actual cortico-cortical connectivity, this nevertheless suggests that there is an intimate link between the spatiotemporal profile of burst suppression and the underlying brain connectivity. This will have to be taken into account when trying to improve the realism of such simulations. Thus, by being more specific about the anatomical structure of our mesoscopic model it may become possible that observations of burst suppression patterns will enable estimation of the effective connectivity of the bursting tissue. This would provide a new window on a difficult to access but key property of the brain.

Our present simulation has been restricted to studying the role that one slow modulatory system might have in the genesis of the burst suppression pattern. However, given the feedback inherent in the physiological and anatomical organization of cortex it is certainly only one of many systems that are capable of modulating cortical excitability, and hence the emergence of fast-slow bursting activity. Indeed we might hypothesize that such slow modulatory systems will span a number of functional scales in the brain—from perturbations in the autoregulatory coupling of neuronal and metabolic activity to alterations in the dynamics of cortico-thalamic and cortico-cortical feedback, to mention only the most obvious. However, regardless of the specifics of the slow system it is clear that any theory purporting to account for the genesis and features of cortical electrodynamics must be able to account for the reversible emergence of burst suppression in response to the action of anesthetic agents. In this respect both our model and the model of Ching et al. (2012) may be seen as meeting this requirement, even though they take as their starting points neuronal activity modeled at different spatial scales. A possible advantage of our approach, besides being able to deal easily with a spatially extended cortex, is that the modeled action of anesthesia is directly coupled to the emergence, and subsequent disappearance, of bursting. In contrast, in Ching et al. (2012) the parameters defining anesthetic action (τ_GABA_ and *g*_GABA_) are not directly related to the parameter *J*_ATP_ (the metabolic production rate of ATP) that defines the emergence of bursting.

Our model of synaptic depression is driven by pre-synaptic firing rates. This is unproblematic for the quasi-instantaneous local activity. However, an issue arises due to the propagation of action potentials from distant sources with finite velocities along cortico-cortical fibers. The current formulation of Equations (31, 33) as to how such distant inputs drive local PSPs is not entirely faithful to the actual physiology: the pre-synaptic firing rate is now modulated with the concurrent synaptic dynamics at the *distant* pre-synaptic site, which is then propagated with conduction delay; whereas it would be more physiologically accurate to propagate the pre-synaptic firing rates, and then modulate these conduction-delayed inputs with *local* synaptic dynamics. However, this would mean tracking separately local synaptic dynamics driven by quasi-instantaneous local (*C*^*S*^_*l*_) and delayed distant (*C*^Φ^_*ek*_) pre-synaptic firing rates, respectively. Our current model requires only one local synaptic dynamics (*C*_*l*_), making it computationally simpler. Furthermore, one can argue that the current formulation better separates distant sources: If there are two distant sources, but only one of them begins to fire at higher rates, then only its signal would become depressed by the resulting synaptic dynamics, but not that of the other. Whereas if we were to drive a local *C*^Φ^_*ek*_ directly with the sum of the delayed signals, then the higher firing of one would lead to synaptic depression also of the other. Yet one can argue to the contrary that such a conflation of distant inputs is simply part of the averaging approximation involved in considering neural populations rather than individual cells. We have made here a choice of convenience, but this may not always be possible. The parameters characterizing the (excitatory) synaptic dynamics are in this work taken to be spatially homogeneous. We are not aware of any extant empirical evidence that would require a significant deviation from homogeneity, but the alternative method mentioned above would be more suited for this case. For as currently formulated, the synaptic dynamics will be determined by parameters located at the origin of such activity and not, as must be physiologically the case, its pre-synaptic termination. A further possibility that we have not considered, due to the lack of any clear empirical data, is that these parameters themselves depend systematically on anesthetic concentration. If such a relationship is demonstrated, then this could act as a possible source of spatial inhomogeneity in the parameters of the synaptic dynamics, under the assumption that anaesthetic action shows spatial variability.

Finally, there are obvious extensions to this work that should be considered, but were beyond the scope of this initial investigation. The most obvious extension is to more systematically consider the effects of variations in the neural field model parameters defining the resting (unperturbed) EEG, and to study the resulting dynamics for spatially homogeneous models of synaptic resource depletion and anesthetic action. Furthermore, the neural field model used here can be extended to an equivalent neural mass mesh constrained by a real cortical head model based on MRI data (Bojak et al., 2010, 2011). Then one could investigate regional variations of the neural mass parameters with areal boundaries defined according to any of the available cortical structural/anatomical atlases [e.g., the Harvard-Oxford cortical and subcortical structural atlas (Desikan et al., 2006), or the Jülich histological atlas (Eickhoff et al., 2005)]. For example, each region could be assigned a distinct parameter set identified as producing physiologically plausible EEG within the physiologically admissible/plausible parameter space (Bojak and Liley, 2005). One could then additionally study regional variations in the synaptic resource depletion and/or anesthetic action parts of the model, as well as the interactions of heterogeneities in the component systems.

A perhaps less immediately obvious development, which however will be necessary to obtain a deeper understanding of the dynamical mechanisms responsible for the emergence of burst-suppression, would be some form of systematic bifurcation analysis. In such an analysis, the slow system would be “frozen”, i.e., one would set the synaptic ∂*C*_*l*_/∂*t* ≡ 0, and a bifurcation analysis of the remaining “fast” subsystem would be performed by treating the *C*_*l*_ as bifurcation parameters. Such a bifurcation analysis, known as a fast-slow analysis, was pioneered by Rinzel (1985) in his formal analysis of bursting in biophysical models of the neuronal action potential. While such a bifurcation analysis is relatively straightforward for the temporal dynamics of non-linear ODE systems, using a variety of available bifurcation software tools like AUTO, Content, and MatCont (Meijer et al., 2013), it is considerably more challenging in systems of non-linear PDEs of the type we have studied here. A fast-slow bifurcation analysis of the spatiotemporal dynamics of burst suppression will require the development of new numerical methods and tools, which are only just beginning to emerge (Green and van Veen, 2014).

Our work then represents only a first step toward a deeper understanding of the spatiotemporal dynamics of burst suppression, in particular as induced by anesthesia. Yet it is already clear from the theoretical results obtained here, which were motivated by the recent experimental results of Lewis et al. (2013), that the classical understanding of burst suppression as spatially homogeneous phenomenon has become outdated. This can only add to the importance of burst suppression as a dynamical probe to investigate the properties and function of cortical tissue, whether in a theoretical modeling or an applied clinical setting. We expect that in the near future theory will be further challenged by the rapid technological advances in electrophysiology and neuroimaging, which are producing increasingly accurate and dense measurements of neuronal activity and cortical dynamics. This hopefully will allow us to test to what extent the synaptic resource depletion mechanism proposed here is indeed the driver of the observed burst suppression dynamics.

## Author contributions

IB and DTJL developed the model. IB and ZVS programmed the simulation software, analyzed the generated data, and produced the figures and movies. IB and DTJL wrote the paper. All authors have approved the final version of the paper and the Supplementary Material.

### Conflict of interest statement

The authors declare that the research was conducted in the absence of any commercial or financial relationships that could be construed as a potential conflict of interest.

## Footnotes

^1^For propofol anesthesia this means that alpha activity present prior to the onset of burst suppression is retained within the bursts (Ching et al., 2012). In contrast, during isoflurane anesthesia slow-wave and delta activity persists during bursts (Kroeger and Amzica, 2007).

^2^We note that as compared to Bojak and Liley (2005), we have here rescaled *v*_*ek*_ and λ_*ek*_ as in Bojak and Liley (2010), so as to remove a factor 3/2, which technically arises from an expansion of an ansatz for the corresponding Green's function.

^3^The last term *W*_−1_[exp(*a*/κ^2^)/(1 − *b*κ)] actually has *b* = −*W*_−1_(−1/*e*^2^) and *a* = −1 + ln(−1 + *b*). It is real for κ ≥ 1 and for κ = 1 becomes −1. To avoid spurious imaginary terms one can set *a* = −0.23630117 for a *b* = 3.1462 of limited accuracy.

## Supplementary material

We include the animations below to illustrate the results described in this work. In all cases the 512 × 512 grid for numerical evaluation has been averaged over 4 × 4 patches, and strong compression and low time sampling may limit the movie quality, see the discussion in Section 2.3.

Movie 1 **Animation showing the *h*_*e*_ dynamics for variations in the isoflurane concentration, for details see Figures 2, 3**. Note that the parts prior and after the emergent burst suppression have been sped up by increasing the time step between the movie frames.

Movie 2 **Animation showing *h*_*e*_ (top panel) and Γ_*ee*_ (bottom panel), respectively, at 0.25 mM isoflurane with an increased *f*_*i*_ = 1.25 inside a circular patch—preventing burst suppression there—while the regular *f*_*i*_ = 0.175 is kept elsewhere**. For further detail see Figure 4 and the text. Note that the induction phase is not shown.

Movie 3 **Animation showing the *h*_*e*_ dynamics at 0.25 mM isoflurane with regular λ_*ek*_ = λ_1_ = 2.7 cm, for detail see Figure 6**. Note that the induction phase is not shown.

Movie 4 **Like Movie 3 but with λ_*ek*_ = λ_2_ = 2.4 cm**.

Movie 5 **Like Movie 3 but with λ_*ek*_ = λ_3_ = 2.1 cm**.
